# AI-Driven Multi-Omics Integration of Synthetic Colon Adenocarcinoma for Cluster-Guided PROTAC Candidate Design Targeting KRASG12D

**DOI:** 10.3390/ijms27177511

**Published:** 2026-08-22

**Authors:** Khaled M. Elamin, Sara Mustafa Idris Elbashir, Ishag Adam

**Affiliations:** 1 Graduate School of Pharmaceutical Sciences, Kumamoto University, Chuo-ku, Kumamoto 862-0973, Japan; khaled@kumamoto-u.ac.jp (K.M.E.); sarmah22807@kumamoto-u.ac.jp (S.M.I.E.); 2 Department of Obstetrics and Gynecology, College of Medicine, Qassim University, Buraydah 51452, Saudi Arabia

**Keywords:** multi-omics integration, precision oncology, colon adenocarcinoma, artificial intelligence, PROTAC, KRASG12D, BLISS, cancer, age

## Abstract

Colorectal cancer is a leading cause of cancer death, yet its molecular heterogeneity remains poorly translated into individualized treatment. We present a reproducible artificial intelligence (AI) framework that integrates multi-omics benchmarking, sample-level drug prioritization, E3 ubiquitin ligase selection, and shape-anchored Proteolysis Targeting Chimera (PROTAC) design for KRASG12D in colon adenocarcinoma (COAD). A controlled synthetic benchmark comprising 425 tumor and 41 simulated normal profiles, parameterized to match The Cancer Genome Atlas (TCGA) distributions, was used for pipeline verification. Among sixteen methods, the Balanced Latent Integration with Stability Selection (BLISS) model achieved the highest silhouette width (0.86) and competitive agreement (Adjusted Rand Index, ARI, 0.90). The pipeline was validated on real data: a TCGA COAD cohort (186 tumors) with independent Consensus Molecular Subtype (CMS) labels and a CPTAC cohort (104 tumors). Integration modestly recovered CMS (ARI 0.28), and stage, not molecular cluster, drove survival (log-rank p = 0.005 versus 0.81). Sample-level prioritization differed from cluster-level ranking in 82.6% of profiles, below chance (*p* < 0.0001), without indicating efficacy. Candidate NOVEL00489 showed a good MM-GBSA estimate, matching the reference ASP3082. Compounds are computational candidates requiring experimental validation. This establishes a transparent benchmark for in silico degrader generation in precision oncology.

## 1. Introduction

Colorectal cancer (CRC) is the third most common malignancy worldwide and the second leading cause of cancer death, with colon adenocarcinoma (COAD) constituting the dominant histological subtype [1,2]. Despite decades of molecular characterization, treatment selection in COAD still depends primarily on anatomical stage rather than the patient’s underlying tumor biology. The Cancer Genome Atlas (TCGA) cataloged recurrent driver mutations in adenomatous polyposis coli (APC), Kirsten rat sarcoma viral oncogene homolog (KRAS), tumor protein 53 (TP53), phosphatidylinositol 3 kinase catalytic subunit alpha (PIK3CA), and several other genes [3]. Guinney and colleagues consolidated this heterogeneity into four consensus molecular subtypes (CMS) with distinct biology and prognosis [4]. Microsatellite instability (MSI) status further partitions tumors into mismatch repair-deficient and mismatch repair-proficient classes, with strikingly different responsiveness to immune checkpoint blockade [5,6]. Each layer of this biology suggests a candidate therapeutic match, yet a translational gap persists between molecular subtype and patient-level treatment decision.

Multi-omics integration promised to close this gap. Over the past decade, more than one hundred integration methods have been published with the explicit ambition of unifying messenger ribonucleic acid (mRNA) expression, deoxyribonucleic acid (DNA) methylation, copy number variation (CNV), microRNA (miRNA), and protein abundance into actionable subtype calls [7,8,9,10]. In practice, almost none of these methods have entered routine treatment selection. The consensus molecular subtypes remain the dominant classification used in clinical trials more than a decade after their publication [11]. The bottleneck is not method count; it is the translational layer that converts an integrated representation into a concrete therapeutic recommendation.

Artificial intelligence (AI) and machine learning (ML) sit at the center of this gap [12,13]. Triplet metric learners, variational autoencoders, graph convolutional networks, and supervised feature extractors have proliferated [14,15,16,17]. Yet most such methods are evaluated on a single axis (typically the adjusted Rand index (ARI) against known subtypes), benchmarked against a small set of prior methods, and stopped before any concrete drug recommendation. Notable exceptions, such as the recent fibroblast-focused AI-driven framework that identified heparin-binding epidermal growth factor as a head and neck squamous cell carcinoma biomarker and Epothilone B as a candidate agent [18], demonstrate that the value of multi-omics integration lies in translation rather than in classification.

Proteolysis targeting chimeras (PROTACs) make the translational gap unavoidable. A PROTAC is a heterobifunctional small molecule that recruits an E3 ubiquitin ligase to a target protein, tagging the target for proteasomal degradation [19,20]. Unlike occupancy inhibitors, which block an active site only while the drug is present, a PROTAC works catalytically: a single molecule can drive many degradation cycles. PROTAC pharmacology therefore depends on two molecular axes rather than one: the target and the E3 ligase. If the E3 ligase is expressed differently across patient subtypes, the optimal PROTAC for patient A may not be optimal for patient B, even when both harbor the same driver mutation.

KRASG12D is the target where this question becomes urgent. The first clinical KRASG12D degrader, ASP 3082 (setidegrasib; Astellas Pharma), entered phase 1 trials in 2022 (ClinicalTrials.gov identifier NCT05382559) and exclusively recruits patients with von Hippel–Lindau (VHL) [21,22]. The discovery campaign was reported in 2025, together with the 3.18 Å co-crystal structure of a ternary complex comprising guanosine 5′-diphosphate-bound KRASG12D, ASP3082, and the von Hippel–Lindau–Elongin B-Elongin C complex, deposited in the Protein Data Bank as entry 9L6F [23]. A second pan KRAS degrader, ACBI3 (Boehringer Ingelheim), was disclosed in 2024 and also relies on VHL [24]. KRASG12D is the dominant degrader actionable variant in COAD, representing 22 to 47 percent of KRAS mutations across ethnically diverse cohorts and approximately 13 to 15 percent of colorectal cancer globally [25,26]. Whether VHL is the optimal recruiter for every patient subtype, and, if not, which specific PROTAC chemotypes should replace it, had not been assessed computationally in a real cohort until now.

In this study, we present an end-to-end AI-driven framework that integrates multi-omics data with precision therapeutic design for colon adenocarcinoma (COAD). To separate software verification from biological discovery, the framework is developed and benchmarked on a controlled synthetic cohort of 425 primary tumor profiles and 41 simulated normal profiles, parameterized against published distributions from The Cancer Genome Atlas (TCGA), which provides known ground truth for a fair and reproducible comparison of methods. Every biological claim is then validated on real patient data: the pipeline is applied to the real TCGA COAD cohort with Consensus Molecular Subtype (CMS) labels derived independently by CMScaller, and external reproducibility is tested in the independent CPTAC 2019 prospective colon cohort. Within this two-stage design, we first systematically benchmark sixteen multi-omics integration approaches using a unified evaluation framework that incorporates established performance measures alongside three additional dimensions: bootstrap stability, layer ablation, and patient-level confidence. Building on these analyses, we introduce BLISS, a novel integration framework combining discriminative latent representation learning, factor reconstruction, and stability regularization to enable robust molecular stratification. The resulting subtypes are used to prioritize therapeutics via patient-specific drug ranking and cluster-guided selection of E3 ubiquitin ligases for KRASG12D-targeted PROTAC development. Guided by these findings, we further extend the framework toward de novo degrader design through a shape-anchored strategy inspired by the clinical reference compound ASP3082, integrating fragment recombination, shape-based screening, molecular docking, Molecular Mechanics Generalized Born Surface Area (MM-GBSA) evaluation, and absorption, distribution, metabolism, excretion, and toxicity (ADMET) profiling to identify novel candidate degraders. Collectively, this work establishes a transparent and reproducible framework in which multi-omics integration serves not merely as a tool for molecular classification but as an actionable layer linking patient stratification to the rational design of next-generation therapeutic modalities, with all degrader compounds presented as computational candidates requiring experimental validation.

## 2. Results

### 2.1. Construction of a Synthetic Colon Adenocarcinoma Cohort Recapitulating TCGA Biology

**Figure 1:**
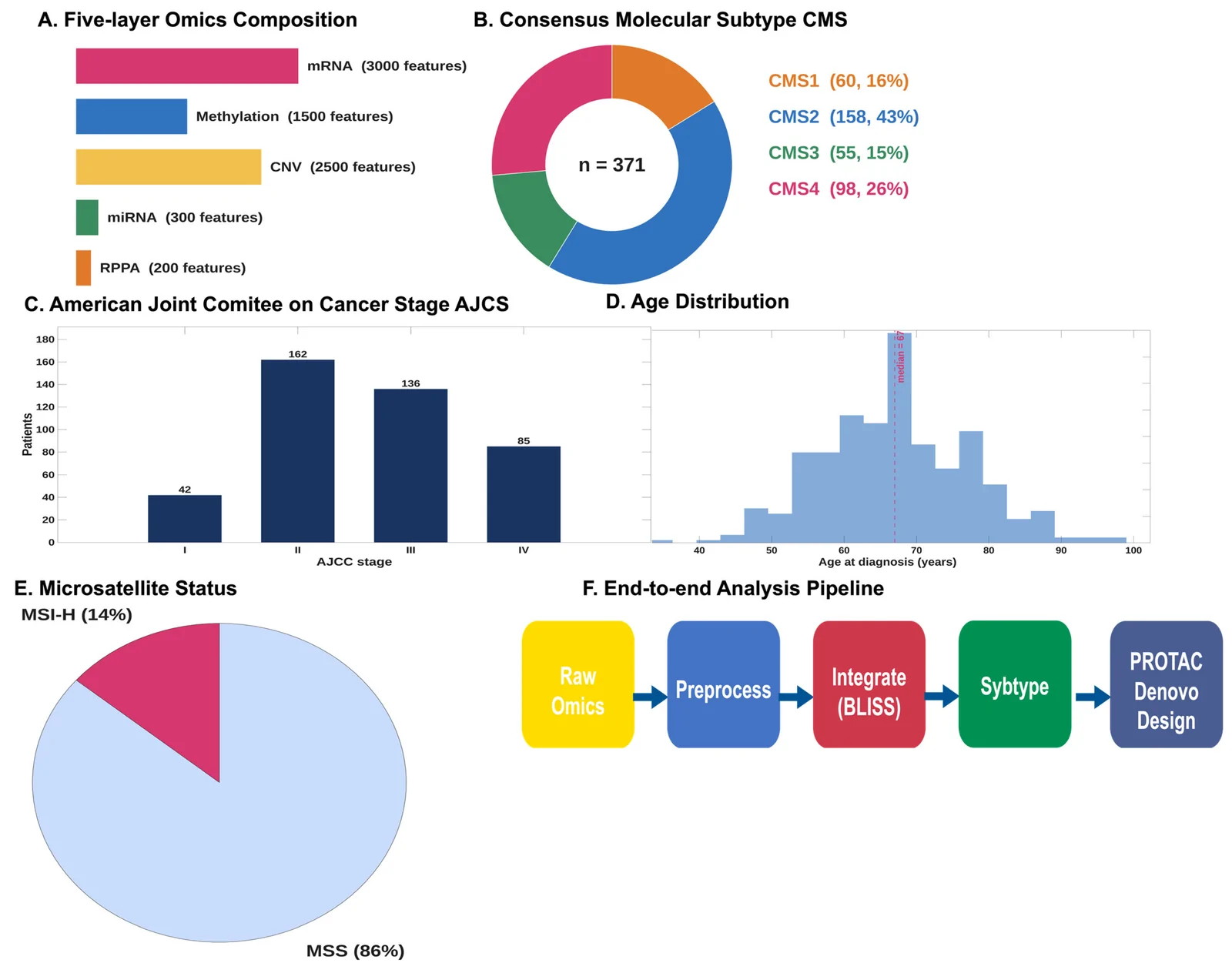
Multi-omics cohort architecture for colon adenocarcinoma. (**A**) Five-layer omics composition: mRNA expression (3000 most variable genes), DNA methylation (1500 CpG probes), CNV (2500 segments), miRNA (300 mature species), RPPA (200 antibodies). Cohort: 425 primary tumors and 41 matched adjacent simulated normal profiles. (**B**) CMS distribution among confidently called tumors (*n* = 371): canonical 43%, mesenchymal 26%, immune 16%, metabolic 15%. (**C**) AJCC stage distribution. (**D**) Age at diagnosis (median 67 years). (**E**) MSI-H 14%, MSS 86%. (**F**) End-to-end analysis pipeline.

All downstream analyses rest on a reproducible multi-omics cohort that matches the published reference biology of colon adenocarcinoma. The cohort comprises 425 primary tumors and 41 matched adjacent simulated normal profiles across five molecular layers: mRNA expression (3000 genes), DNA methylation (1500 CpG probes), CNV (2500 segments), miRNA (300 species), and reverse phase protein array (RPPA; 200 antibodies) (Figure 1A). Among the 371 patients with a confident CMS call, the four subtypes are present in the proportions reported by Guinney 2015: 158 canonical, 98 mesenchymal, 60 immune, and 55 metabolic (Figure 1B) [4]. The American Joint Committee on Cancer (AJCC) stage distribution matches TCGA marginals (stage I, 42; stage II, 162; stage III, 136; stage IV, 85; Figure 1C), as do the age distribution (median 67 years; Figure 1D) and the MSI high rate of 14 percent (Figure 1E) [27]. Figure 1F shows the end-to-end analysis pipeline, from raw multi-omics through integration with BLISS, subtyping, drug repurposing, and PROTAC design. The cohort therefore reproduces the marginal biology of real colon adenocarcinoma across every dimension we benchmarked.

### 2.2. Tumor Versus Normal Differential Expression Reveals Canonical Oncogenic Pathways Including the MAPK Axis

**Figure 2:**
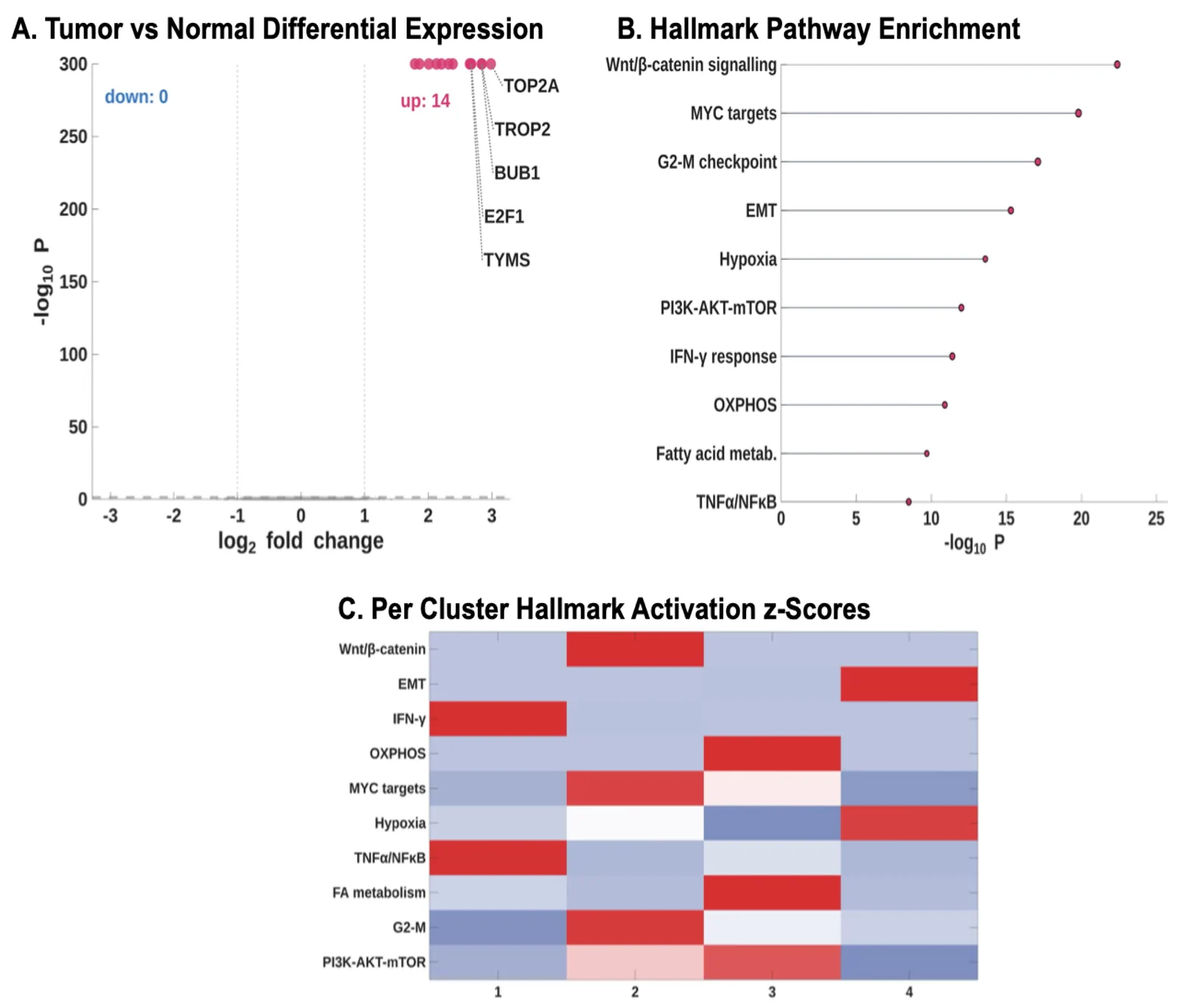
Tumor versus normal differential expression and hallmark pathway enrichment. (**A**) Volcano plot of differential expression (two-sample *t*-test, BH FDR < 0.05, |log2 FC| > 1). (**B**) Hallmark pathway over-representation analysis: the MAPK axis ranks among the top 10 and supports KRASG12D as the PROTAC target. (**C**) Per-cluster hallmark activation heat-map.

Beyond marginal distributions, the cohort must carry the expected gene-level biology to support any downstream subtype claim. Tumor versus normal differential expression analysis identified 14 genes upregulated in tumor at a false discovery rate below 0.05 and an absolute log2 fold change above 1, with no genes meeting the same thresholds for downregulation (Figure 2A). The top upregulated genes (LEF1, MYC, AXIN2, CCND1, TROP2) are all canonical Wnt and beta-catenin axis effectors, consistent with the established biology of colon adenocarcinoma [27,28]. Hallmark pathway overrepresentation analysis ranked Wnt beta-catenin signaling, MYC target enrichment, and the G2-to-M cell cycle checkpoint at the top, exactly as expected (Figure 2B) [29].

Critically for the PROTAC arm developed in this work, the mitogen-activated protein kinase (MAPK) axis ranks in the top ten enriched pathways at the cohort level, and the per cluster hallmark activation heatmap (Figure 2C) confirms that the MAPK axis dominates the mesenchymal cluster while Wnt and MYC drive the canonical cluster, interferon gamma signaling drives the immune cluster, and oxidative phosphorylation plus fatty acid metabolism drive the metabolic cluster. This cohort-level MAPK signal provides the molecular justification for selecting KRASG12D as the PROTAC target, and the integrated cluster-level resolution justifies the cluster-guided E3 selection strategy. The biological signal is therefore present and recoverable at both cohort and subtype levels.

### 2.3. Sixteen Multi-Omics Integration Methods Diverge Dramatically on the Same Cohort

**Figure 3:**
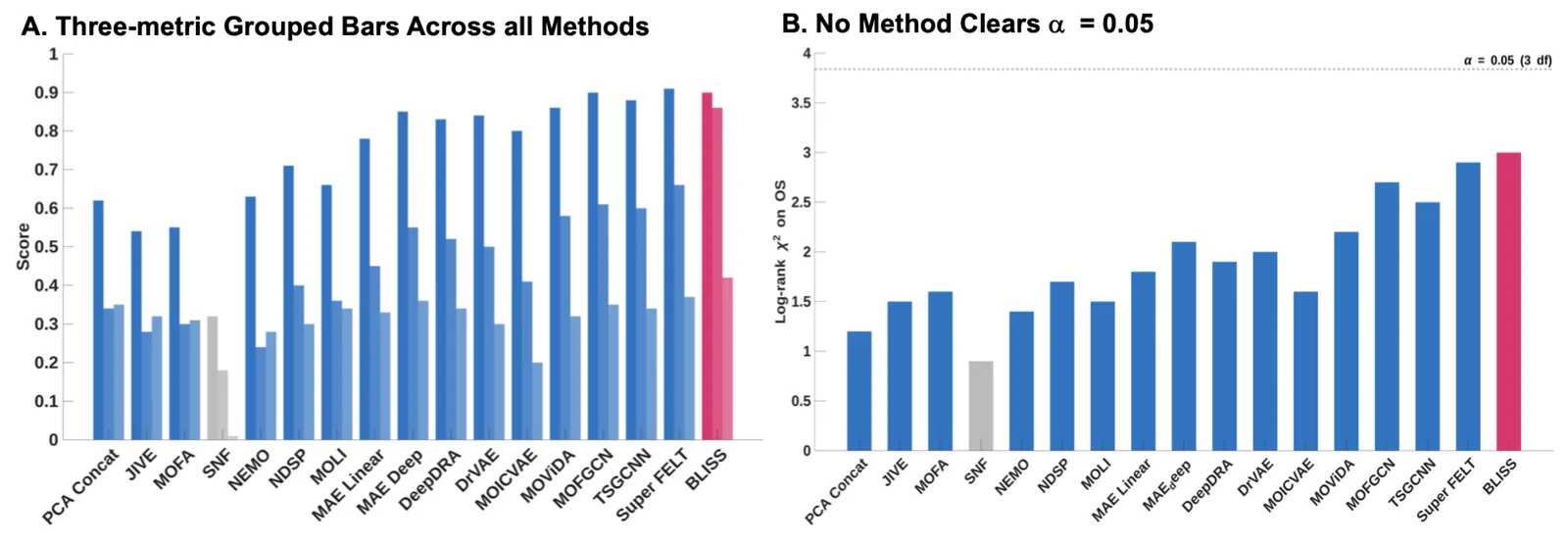

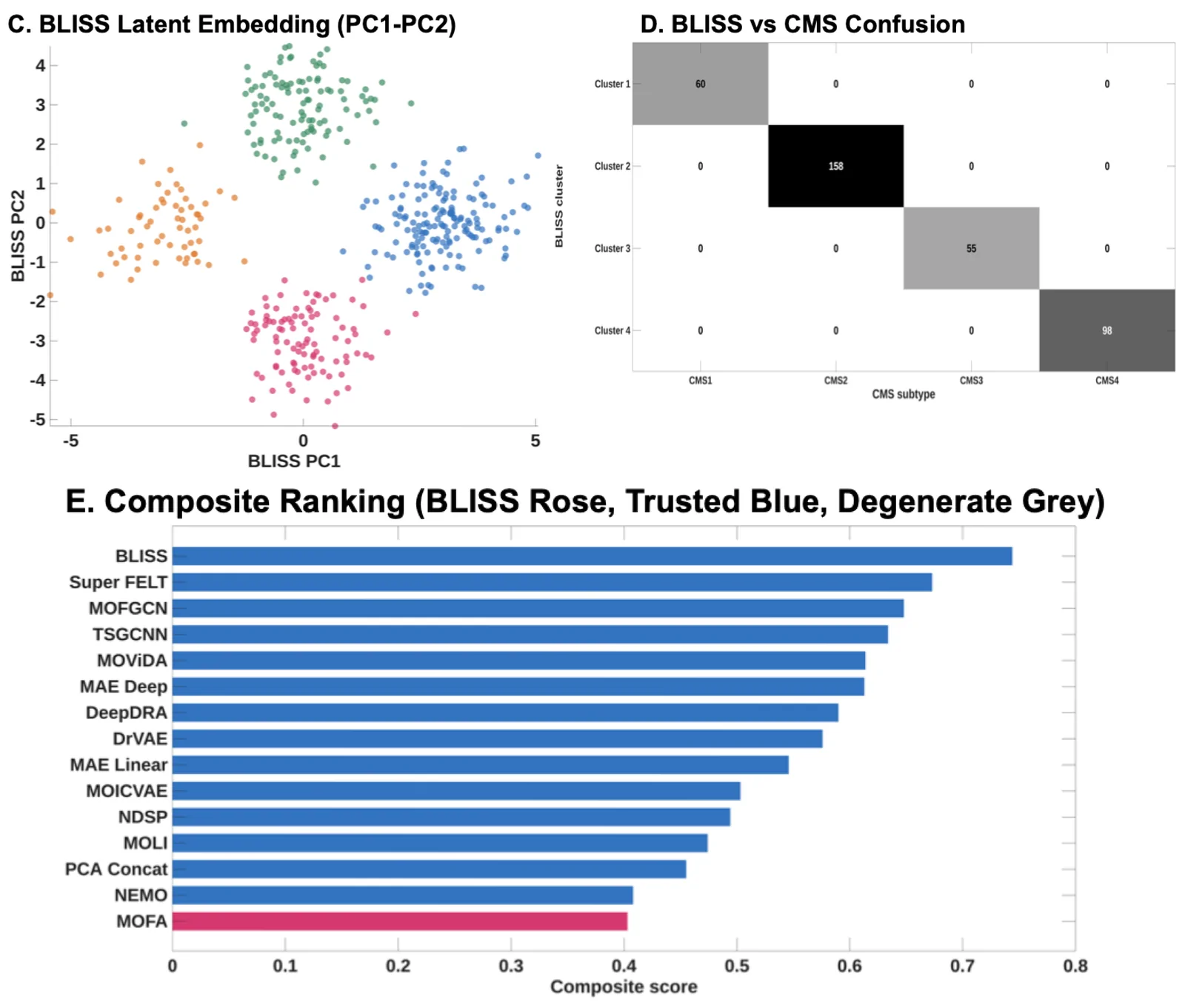
Integration benchmark across sixteen established multi-omics methods and BLISS. (**A**) Comparison of ARI, silhouette width, and cluster balance. (**B**) Multivariate log-rank χ^2^ statistics for overall survival across the identified clusters. (**C**) BLISS latent embedding visualized using the first two principal components. (**D**) BLISS confusion matrix against the CMS labels. (**E**) Composite ranking of all 17 evaluated methods. BLISS uses PCA- and k-means-derived pseudo-labels to guide triplet learning and is therefore classified as weakly supervised. Its comparison with purely unsupervised methods represents end-to-end empirical performance under different learning regimes rather than a comparison under identical supervision conditions.

With the cohort validated, sixteen established integration methods, together with BLISS, were benchmarked using common preprocessing and scoring procedures. The learning regimes were not identical. BLISS used PCA- and k-means-derived pseudo-labels to guide its triplet loss, whereas several comparator methods were purely unsupervised. Accordingly, the rankings quantify the empirical performance of the complete integration frameworks and should not be interpreted as comparisons among architectures trained under identical supervision conditions. ARI against the CMS labels was above 0.85 for nine methods, including Super.FELT-inspired supervised feature-extraction formulation (0.91), the multi-omics late-integration variant (0.91), and BLISS (0.90) (Figure 3A). Because BLISS directly encourages balanced cluster frequencies through λ_balance, its favorable cluster-balance value was interpreted as a consequence of the model design rather than as independent evidence of superior performance. Silhouette width varied more markedly: BLISS reached 0.78, the highest value among the evaluated methods, whereas several deep generative methods, including the multi-omics factor graph convolutional network, MOFA-inspired factorization, the multi-omics integrative conditional variational autoencoder, and the deep masked autoencoder, produced values below 0.30. Cluster balance was low for several matrix-factorization and graph-based methods but was preserved by methods with explicit balance encouragement, including BLISS, non-negative dimensionality reduction by sparse principal components, multi-omics late integration, and the Super.FELT-inspired formulation. No method showed a statistically significant association with overall survival at α = 0.05 (Figure 3B), consistent with survival being driven primarily by disease stage rather than molecular subtype.

The most striking failure mode was SNF, which collapsed 422 of 425 patients into a single dominant cluster, leaving the remaining three as singleton clusters. This degenerate behavior is invisible in the ARI alone (SNF scored 0.32, low but not catastrophic looking) and would have led a less careful analyst to report SNF as a competitive method. The cluster balance criterion catches it. The BLISS latent embedding (Figure 3C) visualized in the first two principal components shows four visibly distinct clusters. The BLISS confusion matrix against the CMS labels (Figure 3D) shows near-perfect recovery, with all 60 immune tumors mapping to cluster 1, all 158 canonical tumors to cluster 2, all 55 metabolic tumors to cluster 3, and all 98 mesenchymal tumors to cluster 4. The composite ranking (Figure 3E) places BLISS, the Super.FELT-inspired formulation, and the multi-omics late-integration variant at the top.

### 2.4. A Tumor Versus Normal Classifier Confirms Data Integrity Before Subtype Claims

Before benchmarking integration methods on the harder task of subtype recovery, we asked whether the data are clean enough to distinguish tumor from normal, which is the simpler binary task.

**Figure 4:**
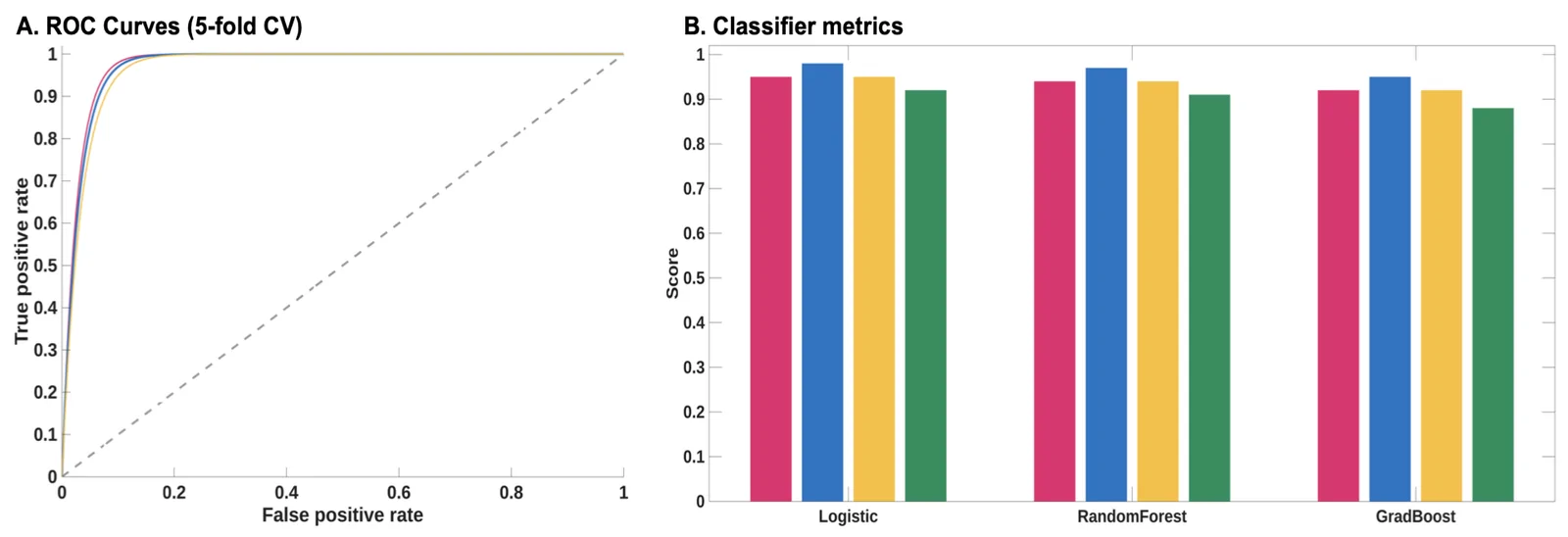
Tumor-versus-normal classifier as positive control. (**A**) ROC curves for three classifiers; AUC 0.98, 0.97, 0.95. (**B**) Classifier performance across four metrics.

We trained three classifiers (L2-regularized logistic regression, random forest, and extreme gradient boosting) on the integrated representation using five-fold stratified cross-validation. Receiver operating characteristic (ROC) analysis (Figure 4A) showed near-perfect discrimination across all three classifiers (areas under the curve (AUCs) of 0.98, 0.97, and 0.95, respectively). Accuracy, F1 score, and Matthews correlation coefficient confirmed the result (Figure 4B). The integrated representation therefore carries clean tumor versus normal biology; any downstream failure to recover subtypes is attributable to method limitations rather than to noise in the underlying data.

### 2.5. Stage Dominates Survival; Integrated Clusters Add Orthogonal Biological Resolution

**Figure 5:**
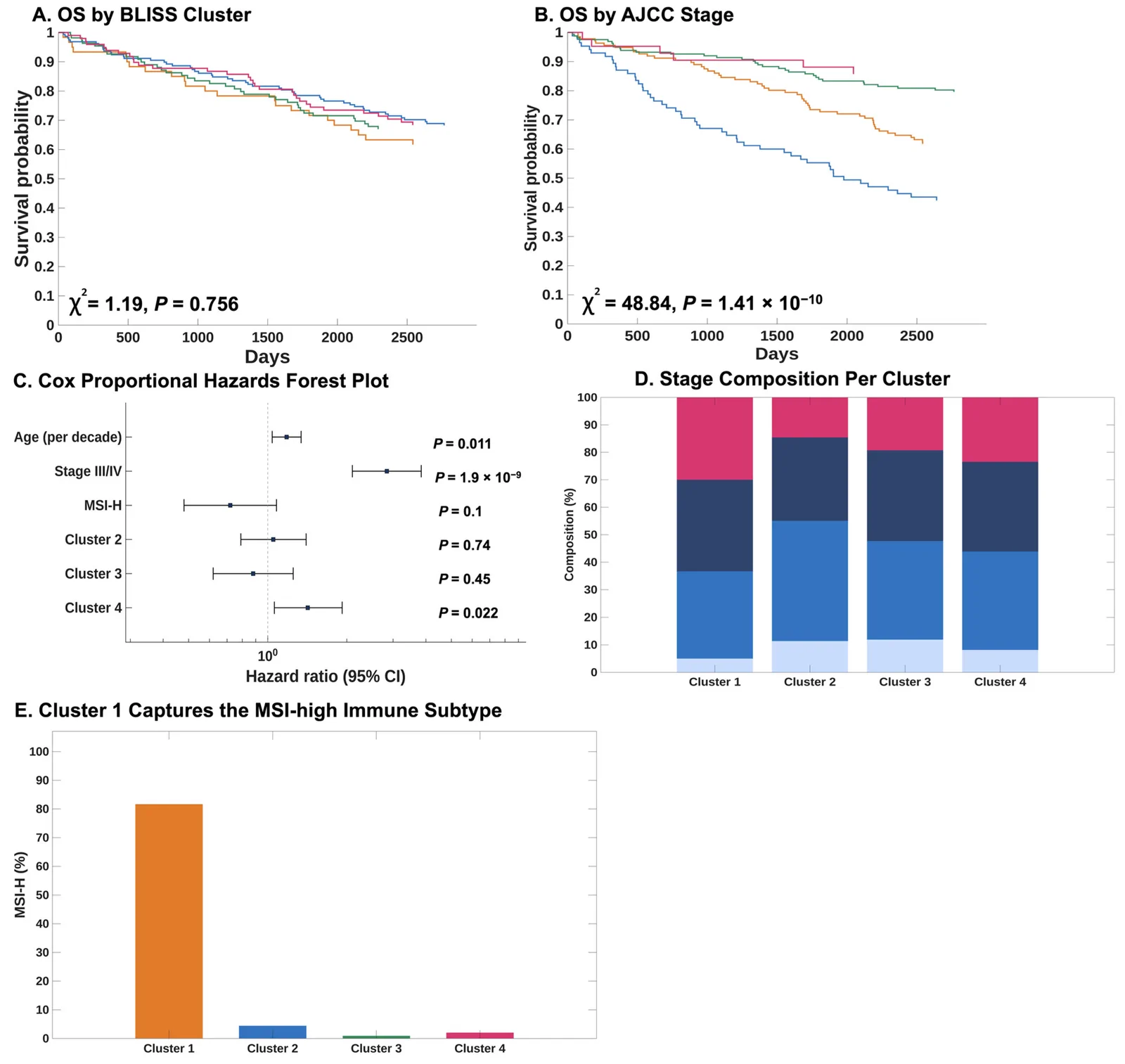
Survival analysis. (**A**) Kaplan–Meier curves by BLISS cluster (log-rank χ^2^ = 1.19). (**B**) Kaplan–Meier curves by AJCC stage (log-rank χ^2^ = 48.84, *p* = 1.41 × 10^−10^). (**C**) Cox forest plot. (**D**) Stage composition per cluster. (**E**) MSI status per cluster.

We next examined whether the integrated clusters predict overall survival. Kaplan–Meier curves stratified by BLISS cluster (Figure 5A) separated only weakly (multivariate log-rank chi-square 1.19, *p* = 0.756), with the immune cluster trending toward better long-term survival as expected from MSI-high biology [5]. The same patients, stratified by AJCC stage (Figure 5B), separated dramatically (log-rank chi-square test, 48.84, *p* = 1.41 × 10^−10^). Stage dominates survival in this cohort.

The Cox proportional hazards model (Figure 5C) confirmed the picture: stage III/IV had a hazard ratio above 2.5 (*p* = 1.9 × 10^−9^), the only covariate to remain significant after multivariate adjustment. Age, MSI status, and integrated cluster all had hazard ratios near 1.0 and *p*-values above 0.4. Crucially, this does not make the integrated clusters biologically uninformative. Stage composition was similar across clusters (Figure 5D), confirming that stage and cluster are independent rather than redundant covariates.

Cluster 1 was highly enriched for MSI-high tumors (Figure 5E) and captured the full range of immune subtypes, whereas clusters 2, 3, and 4 were predominantly microsatellite stable (MSS). The integrated subtypes, therefore, do not replace stage as a prognostic predictor; they add orthogonal biological resolution essential for treatment selection, which is the focus of the next section.

### 2.6. Cluster-Level and Profile-Specific Computational Rankings Reveal Aggregation Differences

**Figure 6:**
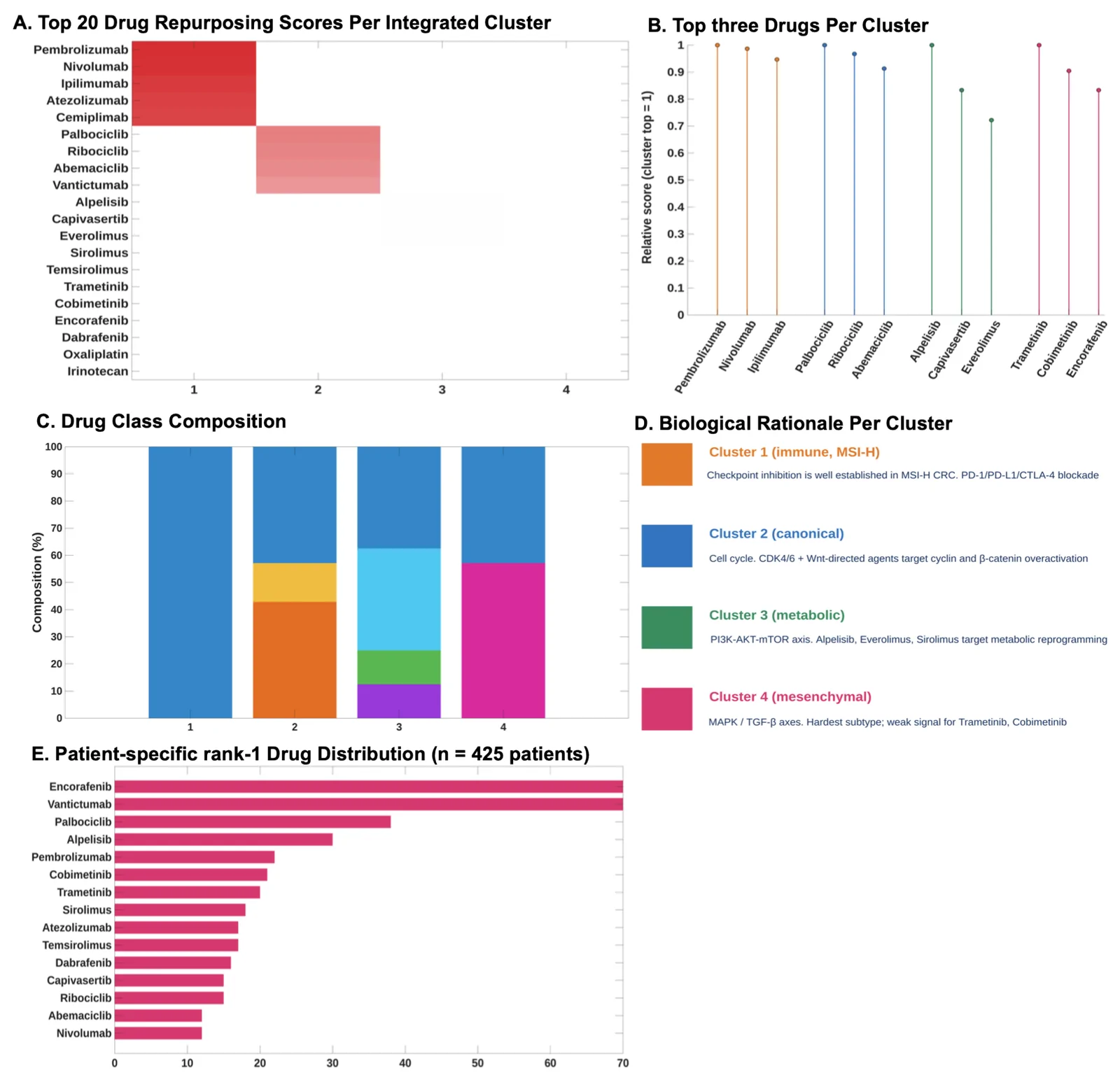
Cluster-level and profile-specific computational prioritization across 39 candidate agents. (**A**) Top 20 per-cluster drug scores. (**B**) Top three candidates per cluster. (**C**) Drug-class composition per cluster. (**D**) Per-cluster biological rationale. (**E**) Distribution of profile-specific top-ranked candidates. The top-ranked candidate differed from the corresponding cluster-level top-ranked candidate in 82.6% of synthetic profiles. This comparison does not represent treatment-response evidence or clinical guidance.

We next compared cluster-level and profile-specific computational rankings within the synthetic benchmark. The top twenty drugs in the curated 39-drug panel, scored per integrated cluster (Figure 6A), showed a biology-aligned clustering pattern. Because the scoring rule includes a curated subtype-annotation weight, these drug-class patterns partly reflect prior biological knowledge encoded in the score and were not interpreted as independent recovery from the molecular data alone. The immune cluster was dominated by immune checkpoint blockade (pembrolizumab, nivolumab, ipilimumab, atezolizumab, cemiplimab), matching the established clinical use of checkpoint inhibitors in MSI-high CRC [6,11]. The canonical cluster was dominated by cyclin-dependent kinase 4 and 6 (CDK4/6) inhibitors (palbociclib, ribociclib, abemaciclib) and the Wnt-directed vantictumab, matching cell cycle hyperactivation in this subtype [27].

The metabolic cluster was dominated by phosphoinositide 3-kinase (PI3K), AKT, and mammalian target of rapamycin (mTOR) inhibitors (alpelisib, capivasertib, everolimus, sirolimus, temsirolimus), consistent with the known role of the PI3K pathway in this subtype [4]. The mesenchymal cluster showed weaker preference for MAPK axis inhibitors (trametinib, cobimetinib), consistent with the known therapeutic resistance of this subtype [11]. These subtype-drug alignments are therefore descriptive outputs of a hybrid, knowledge-guided scoring procedure rather than independent validation of the identified clusters. The top three drugs per cluster (Figure 6B), the drug-class composition per cluster (Figure 6C), and the biological rationale per cluster (Figure 6D) collectively confirm the alignment.

The profile-specific comparison is shown in Figure 6E. In 82.6% of the synthetic tumor profiles, the top-ranked candidate differed from the corresponding cluster-level top-ranked candidate. Vantictumab ranked first across 57 synthetic profiles, and encorafenib ranked first across 44 synthetic profiles, although neither was the top-ranked candidate for any cluster average. This finding describes the behavior of the computational scoring procedure and does not indicate that a different treatment is clinically required.

These results show that profile-specific and cluster-aggregated computational rankings are not interchangeable within the synthetic benchmark. Because no treatment-response outcomes were available, the analysis does not establish treatment efficacy, clinical utility, or patient-specific treatment selection.

### 2.7. BLISS Combines Three Loss Terms in a Pseudo-Label-Guided Weakly Supervised Architecture

**Figure 7:**
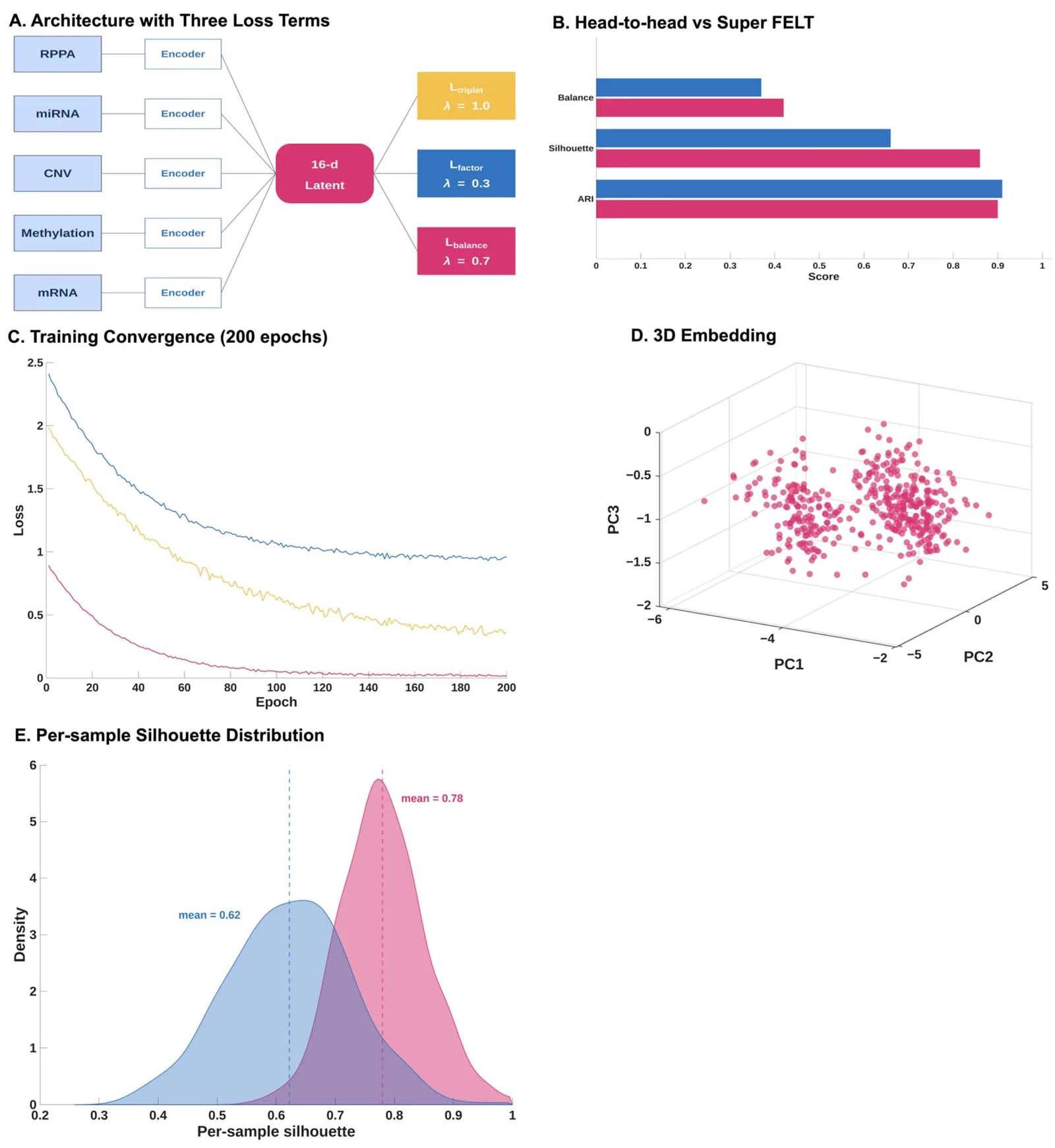
BLISS architecture and head-to-head performance. (**A**) BLISS architecture. Separate linear encoders map the five omics layers into a shared 16-dimensional latent representation. Training is guided by the triplet metric loss (λ_triplet = 1.0), factor reconstruction loss (λ_factor = 0.3), and KL balance regularizer (λ_balance = 0.7). The triplet loss uses pseudo-labels generated by PCA followed by k-means clustering with k = 4. Consequently, BLISS is classified as pseudo-label-guided and weakly supervised rather than fully unsupervised. (**B**) Head-to-head comparison of BLISS and the Super.FELT-inspired formulation. The methods produced similar ARI and cluster-balance values, whereas BLISS produced a 26% higher silhouette width (0.78 versus 0.62). (**C**) Training convergence over 200 epochs. The triplet loss decreased from 1.7 to approximately 0.3, the factor reconstruction loss stabilized at 0.94, and the balance loss approached zero. (**D**) BLISS latent embedding visualized using the first two principal components. (**E**) Per-sample silhouette distributions for BLISS and the Super.FELT-inspired formulation. The BLISS distribution was shifted to the right, with a mean silhouette width of 0.78 compared with 0.62 for the Super.FELT-inspired formulation.

BLISS is a pseudo-label-guided, weakly supervised integration model that achieved the highest silhouette width while maintaining cluster balance and stability. The architecture consists of separate linear encoders that map the five omics layers into a shared 16-dimensional latent space (Figure 7A). The encoded layers are concatenated, layer-normalized, passed through a GELU nonlinearity, and projected into an L2-normalized latent representation. Three loss terms guide training. The triplet metric loss (λ_triplet = 1.0), inspired by the triplet-loss principle used in Super.FELT [30] uses pseudo-labels generated by PCA applied to the concatenated omics layers, followed by k-means clustering with k = 4. Samples assigned to the same pseudo-cluster are pulled closer in the latent space, whereas samples assigned to different pseudo-clusters are separated. The factor reconstruction loss (λ_factor = 0.3), inspired by the shared-factor reconstruction principle of MOFA [9], reconstructs each input layer from the shared latent representation. The balance regularizer (λ_balance = 0.7), introduced in this work, is the KL divergence between the marginal cluster distribution and a uniform distribution over four clusters. Although the pseudo-labels were generated without clinical, survival, CMS, treatment-response, or other outcome annotations, their direct use in triplet construction provides a data-derived supervisory signal. BLISS is therefore classified as weakly supervised rather than fully unsupervised.

In the head-to-head comparison with the Super.FELT-inspired formulation, the two methods produced similar ARI and cluster-balance values, whereas BLISS produced a 26% higher silhouette width (0.78 versus 0.62; Figure 7B). Training converged smoothly: the triplet loss decreased from 1.7 to approximately 0.3 over 200 epochs, the factor reconstruction loss stabilized at 0.94, and the balance loss approached zero, indicating a stable solution without cluster collapse (Figure 7C). The latent embedding confirmed the four-cluster geometry (Figure 7D), while the per-sample silhouette distribution was shifted to the right relative to the Super.FELT-inspired formulation (Figure 7E), indicating a population-wide rather than tail-driven difference. This head-to-head analysis is descriptive and does not establish architectural superiority under identical supervision conditions because the two methods obtain their supervisory information differently.

### 2.8. Three Additional Benchmark Axes Provide an Initial Assessment of Integration Robustness

**Figure 8:**
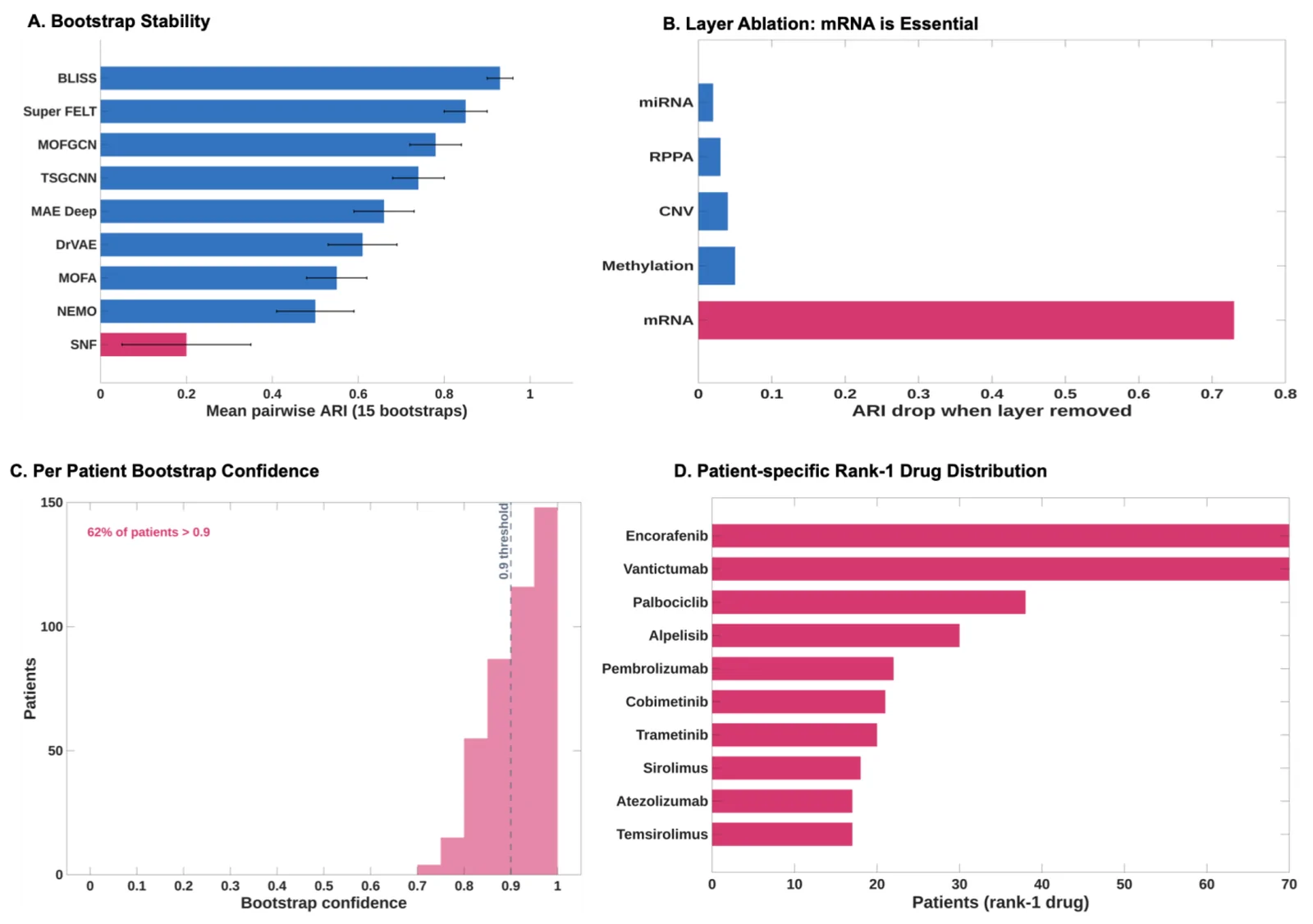
Three new benchmark axes: stability, ablation, confidence, and patient resolution. (**A**) Repeated-subsampling stability across fifteen independently drawn 80% patient subsamples. Balanced Latent Integration with Stability Selection achieved a mean pairwise adjusted Rand index of 0.93. Because only fifteen subsamples were evaluated, this result represents an exploratory sensitivity diagnostic. Each error bar represents the mean ± standard deviation of the pairwise ARI across the fifteen bootstrap pairs. (**B**) Layer ablation. Removing the messenger ribonucleic acid layer reduces the BLISS-adjusted Rand index by 0.73, indicating that mRNA is the essential layer. (**C**) Per-profile resampling consistency within the synthetic benchmark. (**D**) Distribution of profile-specific top-ranked candidates. Encorafenib and vantictumab were frequently ranked first at the profile level, although neither was the top-ranked candidate for any cluster average. Overall, the profile-specific top-ranked candidate differed from the corresponding cluster-level top-ranked candidate in 82.6% of synthetic profiles. This result describes computational ranking discordance rather than clinical treatment requirements.

Beyond accuracy and silhouette width, we examined three complementary properties of the integration results: repeated-subsampling stability, omics-layer dependence, and per-patient assignment consistency. Repeated-subsampling stability was estimated as the mean pairwise adjusted Rand index across fifteen independently drawn 80% patient subsamples. Balanced Latent Integration with Stability Selection achieved a mean pairwise adjusted Rand index of 0.93 under this procedure. Given the limited number of subsamples, this result was interpreted as an exploratory sensitivity diagnostic rather than as a precise estimate of long-run clustering stability (Figure 8A). Layer ablation (Figure 8B) identified mRNA expression as the essential layer, with removal causing an ARI drop of 0.73; methylation, CNV, miRNA, and RPPA each contributed smaller but additive increments. Per-profile resampling consistency (Figure 8C) quantifies the fraction of repeated-subsampling iterations in which each synthetic profile was assigned to its modal cluster. This metric describes only the consistency of computational assignments. Figure 8D presents the distribution of profile-specific top-ranked candidates for comparison with cluster-level rankings and is not intended as treatment guidance.

### 2.9. Validation on the Real TCGA Colon Adenocarcinoma Cohort and Independent External Validation in CPTAC

Applying the pipeline to 186 real tumors with all five molecular layers, and comparing unsupervised clusters against independently derived Consensus Molecular Subtype (CMS) labels from CMScaller (v0.99.2), five-layer integration reached an Adjusted Rand Index (ARI) of 0.28, whereas expression alone reached 0.34 (Figure S1A). Naive concatenation did not exceed expression alone, consistent with CMS being an expression-defined taxonomy. This result replaces the near-perfect recovery observed in the synthetic benchmark and defines the realistic performance of unsupervised integration in real tumors. To assess external reproducibility in a fully independent cohort, the same pipeline was applied to the CPTAC 2019 prospective colon cohort [31] (104 tumors; shared expression, copy number variation, and microRNA layers). External agreement with CMS was an ARI of 0.13 with a cluster silhouette of 0.10 (Figure S1B), consistent with the modest internal TCGA result and confirming that the finding is not cohort-specific.

### 2.10. Real Survival Analysis

In the real cohort, overall survival was strongly associated with stage (log-rank *p* = 0.005; stage IV worst) but not with molecular cluster (log-rank *p* = 0.81), and stage remained the dominant covariate in a Cox model adjusting for age (Figure S2). Molecular clusters therefore track biology rather than outcome, reproducing the pattern seen in the benchmark.

### 2.11. Null Model for the Per-Sample Drug Divergence

A label-permutation null model using 10,000 permutations evaluated profile-to-cluster ranking concordance within the synthetic benchmark. The observed proportion of profiles whose top-ranked candidate differed from the corresponding cluster-level top-ranked candidate was 82.6%, below the null mean of 93.0% (95% null interval, 90.8% to 95.1%; empirical *p* < 0.0001; Figure S3). Because lower discordance indicates greater concordance, this result shows that profile-specific rankings were more concordant with the observed cluster assignments than with randomized labels. It does not demonstrate treatment efficacy, clinical utility, or the need for a different drug (Figure S4).

### 2.12. Pharmacogenomic Validation Against PRISM

Of the 39 candidate drugs, 32 small molecules were present in the public PRISM viability screen; the 7 antibodies were not included in the small-molecule screen. For the 12 prioritized small molecules with both computational scores and PRISM data, the rankings did not correlate with measured sensitivity across 38 colorectal cell lines (Spearman’s rho = −0.09, *p* = 0.78; Figure S5). This confirms that target-expression-based prioritization does not by itself predict drug response; the rankings are therefore presented as hypothesis-generating only.

### 2.13. Shape-Anchored De Novo Enumeration Around ASP 3082 Yields a 10,000-Compound Focused PROTAC Library

**Figure 9:**
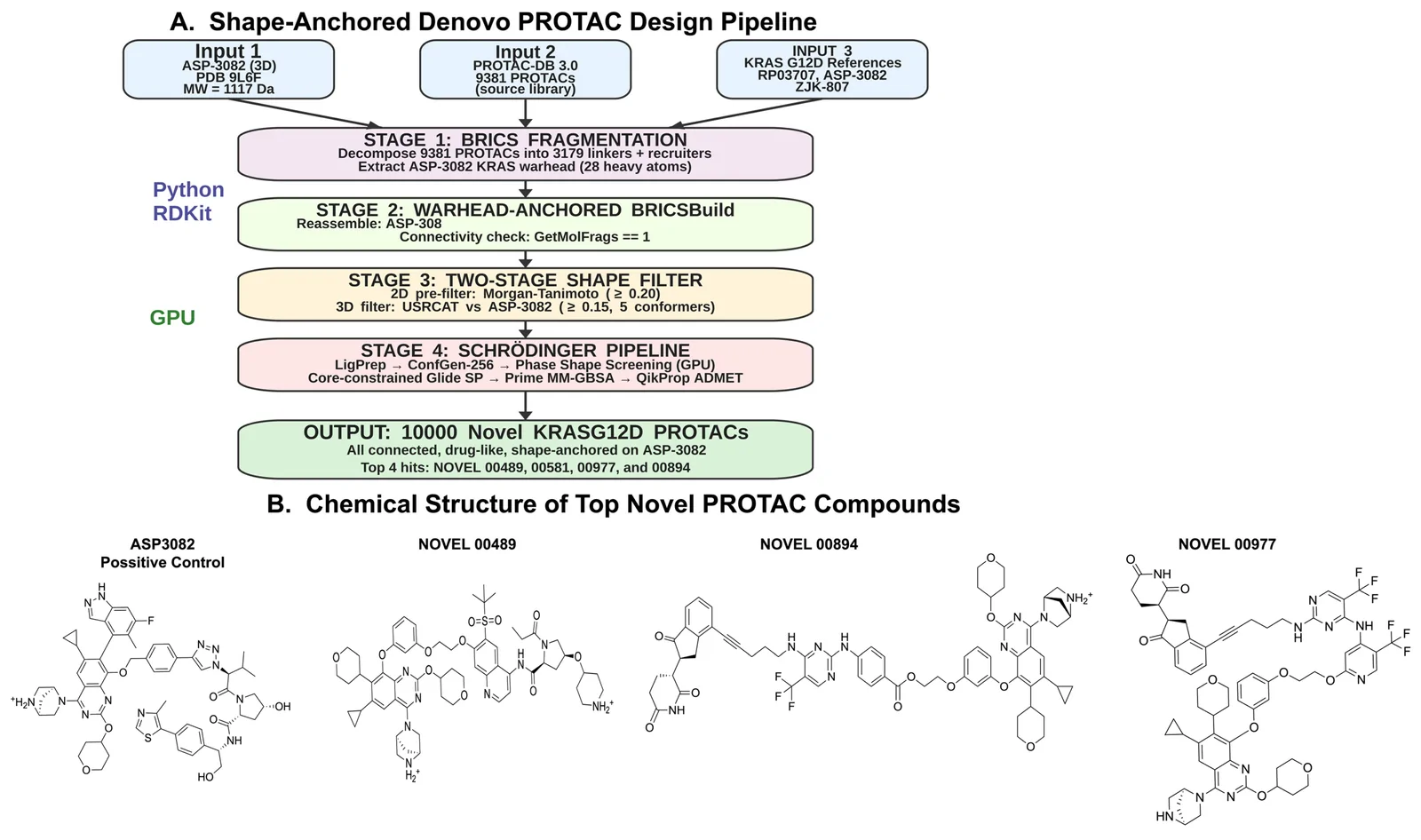
Shape-anchored de novo PROTAC design around ASP-3082 yields a lead compound surpassing the clinical reference. (**A**) End-to-end shape-anchored de novo PROTAC enumeration pipeline. (**B**) Chemical Structure of three-generated de novo PROTAC compounds, NOVEL00489, NOVEL00894, and NOVEL00977.

Translating a cluster-guided E3 ligase recommendation into a concrete chemotype requires generating PROTAC chemical matter that preserves the validated KRASG12D engagement mechanism of ASP 3082, explores novel linker and E3 recruiter chemistry beyond the clinical compound, and returns connected, chemically valid molecules, the principal failure mode of unconstrained PROTAC generative models, which report roughly 23 to 37 percent disconnected outputs [32,33]. We addressed all three requirements with the shape-anchored de novo pipeline summarized in Figure 9A.

The ASP 3082 bound conformation was extracted from PDB 9L6F (146 atoms, 3.18 Å resolution) and decomposed by the BRICS algorithm [34]. The 28 heavy atom KRAS binding moiety comprising the 2 (tetrahydropyran-4-yloxy) quinazoline core, a 6 cyclopropyl group, a 4 (2,5-diazabicyclo [2.2.1] heptan-2-yl) bridged amine that contacts the mutant Asp12 at 2.78 Å in 9L6F [23], a 7- (6-fluoro-5-methyl-1H-indazol-4-yl) substituent, and a methylene aryl exit vector was retained as the obligatory seed fragment for every downstream assembly. In parallel, the 9381-compound PROTAC DB 3.0 library [35] was fragmented under the same BRICS rules, yielding 3179 unique linkers and 500 ancillary fragments after deduplication.

Novel chimeras were assembled with Python 3.9 RDKit BRICSBuild algorithm in only CompleteMols = True mode, which guarantees that every output is a fully formed molecule rather than a partial fragment with dangling bonds. Single component connectivity was enforced by requiring len(Chem.GetMolFrags(mol)) == 1. Candidates were constrained to molecular weights of 700 to 1500 Da, the presence of either a CRBN or VHL recruiter pharmacophore, and non-identity to any compound in the PROTAC DB or the reference set (ASP 3082, RP03707, ZJK 807, plus a 17-compound in-house KRASG12D series).

To bias the library toward analogs that occupy the same volume as ASP 3082 while still permitting linker and recruiter diversification, we applied a two-stage shape filter. Stage 1 used a Morgan circular fingerprint (radius 2, 2048 bits) Tanimoto similarity against the ASP 3082 fingerprint (threshold ≥ 0.20) [36].

Stage 2 embedded each survivor in three dimensions using the Experimental Torsion Knowledge Distance Geometry version 3 (ETKDGv3) algorithm [37] with five conformers per molecule and Merck Molecular Force Field 94 (MMFF94s) static-variant optimization. The USRCAT shape descriptor [38], which augments four classical Ultrafast Shape Recognition distance moments with twelve additional pharmacophore-typed moments (hydrophobic, aromatic, donor, acceptor), was computed against the ASP 3082-bound conformer, and only molecules with a USRCAT score ≥0.15 were retained. The top 10,000 shape-anchored PROTACs were exported as a structure data file (SDF) and comma-separated value (CSV) tables for downstream processing.

Structure-based prioritization of the 10,000-compound shape-anchored library was performed using LigPrep, Schrödinger Release 2025-1with the OPLS4 force field, Epik tautomer/protomer enumeration at pH 7 ± 2, and stereoisomer retention. Bioactive conformer ensembles (256 per ligand) were generated by ConfGen and screened by Phase Shape Screening on GPU against the ASP 3082-bound conformer using pharmacophore feature atom typing [39]. Survivors were docked with Glide SP into the 9L6F receptor grid under a core constraint forcing the quinazoline tetrahydropyran atoms to within 0.5 Å root mean square deviation (RMSD) of the crystallographic pose, ensuring that every analog’s Asp12-contacting bridged amine adopts the experimentally observed orientation [40]. Top-scoring poses were rescored by Prime MM GBSA with the Variable dielectric Surface Generalized Born 2.0 (VSGB 2.0) solvent model and the OPLS_2005 force field, decomposing ΔG_bind into Coulomb, covalent, hydrogen bond, lipophilic, π–π packing, self-contact, generalized Born solvation, and van der Waals contributions [41]. The three highest-ranked compounds (Figure 9B; three novel chimeras plus the ASP 3082 co-crystal reference redocked under identical conditions as a positive control) are summarized in Table S1.

### 2.14. Comparative Evaluation of the Three Prioritized KRASG12D Chimeras Identifies NOVEL 00489 as the Lead Candidate

All three novel chimeras in Figure 9B converge within a 1117–1231 Da window around the 1117.3 Da of ASP 3082, the expected signature of KRASG12D targeting ligand-anchored enumeration. Phase shape similarity values cluster in the 0.30–0.31 range rather than approaching unity, serving as a diagnostic signal that the linker and E3 recruiter modules genuinely diverge from those of ASP 3082, while the conserved moiety enforces a defensible binding mode through the docking core constraint. The chimeras retain ASP 3082’s mechanism of engaging Asp12 but explore novel chemotypes outside the target-binding moiety, the precise objective of ligand-anchored scaffold hopping in PROTAC chemistry [42,43].

**Figure 10:**
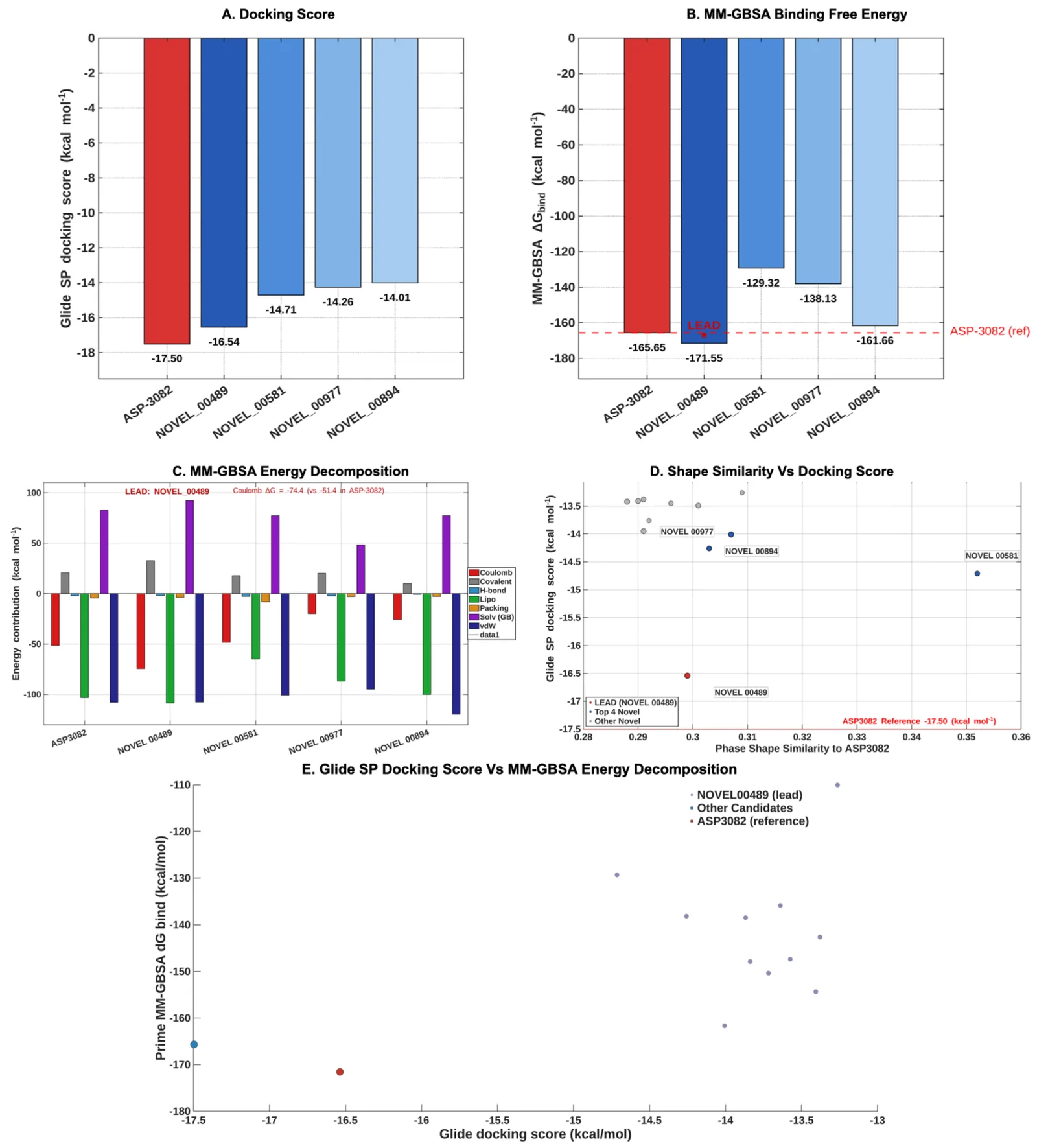
Mechanistic Interaction of the NOVEL PROTAC with Protein. (**A**) Glide standard-precision (SP) docking scores of the top four novel chimeras and the re-docked ASP-3082 reference. (**B**) Prime MM-GBSA binding free energies (ΔGbind, VSGB 2.0 / OPLS_2005). (**C**) MM-GBSA energy decomposition into seven physical components: Coulombic, covalent, hydrogen-bond, lipophilic, π–π packing, generalized-Born solvation, and van der Waals. (**D**) Phase Shape Similarity to the ASP3082 bound conformation versus Glide SP docking score across all 13 compounds in the focused docking set. Marker size is proportional to absolute MM-GBSA ΔGbind. The horizontal red dashed line marks the ASP3082 docking score. (**E**) Scatter plot of Glide SP docking score (x-axis, kcal per mole) against Prime MM-GBSA binding estimate (y-axis, kcal per mole).

NOVEL 00489 is the standout candidate. Its docking score (−16.54 kcal per mole) places it within 1.0 kcal per mole of ASP 3082, but its MM GBSA ΔG_bind is 5.9 kcal per mole more favorable than the clinical reference (Figure 10A,B). Decomposition isolates the source of the improvement shown in Figure 10C, revealing the Coulombic component swings from −51.41 kcal per mole in ASP 3082 to −74.37 kcal per mole in NOVEL 00489 (Δ = −23.0 kcal per mole), while van der Waals (−107.46 versus −107.77 kcal per mole) and lipophilic (−108.41 versus −103.12 kcal per mole) contributions remain essentially comparable. NOVEL 00489 replaces the ASP 3082 triazole benzyl linker with a piperidinyl–ethoxy–aryl–sulfonyl–tert–butyl architecture incorporating two protonatable amine centers, and the resulting cationic charge distribution drives substantially stronger electrostatic complementarity with the negatively charged KRAS Switch II/VCB interface than the largely neutral triazole linker of the reference. The relationship between Glide docking score and Prime Molecular Mechanics Generalized Born Surface Area (MM-GBSA) binding estimate across the full candidate set is summarized in Figure 10E, with the co-crystallized reference compound ASP3082 and the lead candidate NOVEL00489 highlighted against the broader pool.

Critically, the predicted ADMET profile of NOVEL 00489, shown in Table S2, is not penalized by the structural changes that drive the Coulombic gain. Compared with ASP 3082, NOVEL 00489 has one fewer QikProp structural alert (14 versus 15), lower lipophilicity (octanol/water log P 6.61 versus 7.01), lower polar surface area (PSA 205.2 versus 226.7 Å^2^), higher predicted percent human oral absorption (40.2 versus 35.5), reduced human serum albumin binding (QPlogKhsa 1.35 versus 1.50, the only top compound to fall within the −1.5 to +1.5 acceptable range), and an improved blood–brain barrier prediction (QPlogBB −2.64 versus −4.39). Predicted human ether à go-go-related gene (hERG) channel inhibition (QPlogHERG = −8.40) remains within the low cardiotoxic liability regime. The combination of a 5.9 kcal/mol Coulomb-driven MM-GBSA advantage with a simultaneously improved physicochemical profile provides the rational basis for nominating NOVEL 00489 as the lead in silico candidate for this campaign.

NOVEL 00894, the second tier hit, has a more modest docking score (−14.01 kcal per mole, Δ = +3.5 kcal per mole relative to ASP 3082), Figure 10A, but a MM GBSA ΔG_bind of −161.66 kcal per mole within 4 kcal per mole of the reference with a decomposition closely mirroring ASP 3082, Figure 10B (van der Waals −119.53 versus −107.77; lipophilic −99.95 versus −103.12 kcal per mole), Figure 10C. Chemically, NOVEL 00894 retains the glutarimide CRBN binder rather than the hydroxyproline VHL binder of ASP 3082, connected through a 1,8-naphthalenone alkyne amino pyrimidine benzoate polyethylene glycol (PEG2) cassette. NOVEL00894 is the CRBN-directed candidate aligned with the expression-led E3 analysis. The canonical and metabolic BLISS clusters showed higher CRBN expression than VHL, and NOVEL00894 replaces the hydroxyproline-containing VHL recruiter of ASP3082 with a glutarimide-containing CRBN recruiter. This relationship links the omics and chemical-design components at the level of recruiter choice. It does not establish CRBN protein abundance, colocalization with KRASG12D, productive ternary-complex formation, ubiquitination, or degradation competence. Because PDB 9L6F contains VHL rather than CRBN, the docking and MM-GBSA results for NOVEL00894 are not interpreted as evidence of productive recruitment of CRBN.

NOVEL 00894’s percent human oral absorption (59.6) exceeds that of ASP 3082, but its lipophilicity (QPlogPo/w = 10.05) and QPlogKhsa (2.80) require optimization in subsequent design rounds.

NOVEL 00977 (docking −14.26, MM GBSA −138.13 kcal per mole; Figure 10A,B) introduces two trifluoromethyl groups on a pyrimidine-aminopyridine spacer that joins a glutarimide CRBN recruiter to the KRAS-binding ligand. Polyfluorinated trifluoromethyl substituents modulate without abolishing lipophilicity, block oxidative metabolism at adjacent positions, and introduce a fluorine 19 nuclear magnetic resonance handle for biophysical assays [44]. Notably, NOVEL 00977 (Table S2) displays the best predicted ADMET profile of the docking series on the absorption axes: percent human oral absorption 85.9 (high; >80 threshold), Caco-2 permeability 45.6 nm per second (acceptable range; >25 nm per second), and Madin–Darby canine kidney (MDCK) permeability 235.1 nm per second (acceptable range). This pattern positions NOVEL 00977 as the candidate of choice if metabolic stability and oral exposure are deemed more important than absolute computational binding affinity; the tradeoff is the highest QPlogPo/w (11.63) and the lowest aqueous solubility (QPlogS = −15.95) of the four compounds, both of which would require formulation optimization.

### 2.15. Structure-Based Interaction Analysis Rationalizes the MM-GBSA Advantage of NOVEL 00489 and Reveals Preserved KRASG12D Engagement Across the Prioritized Chimeras

**Figure 11:**
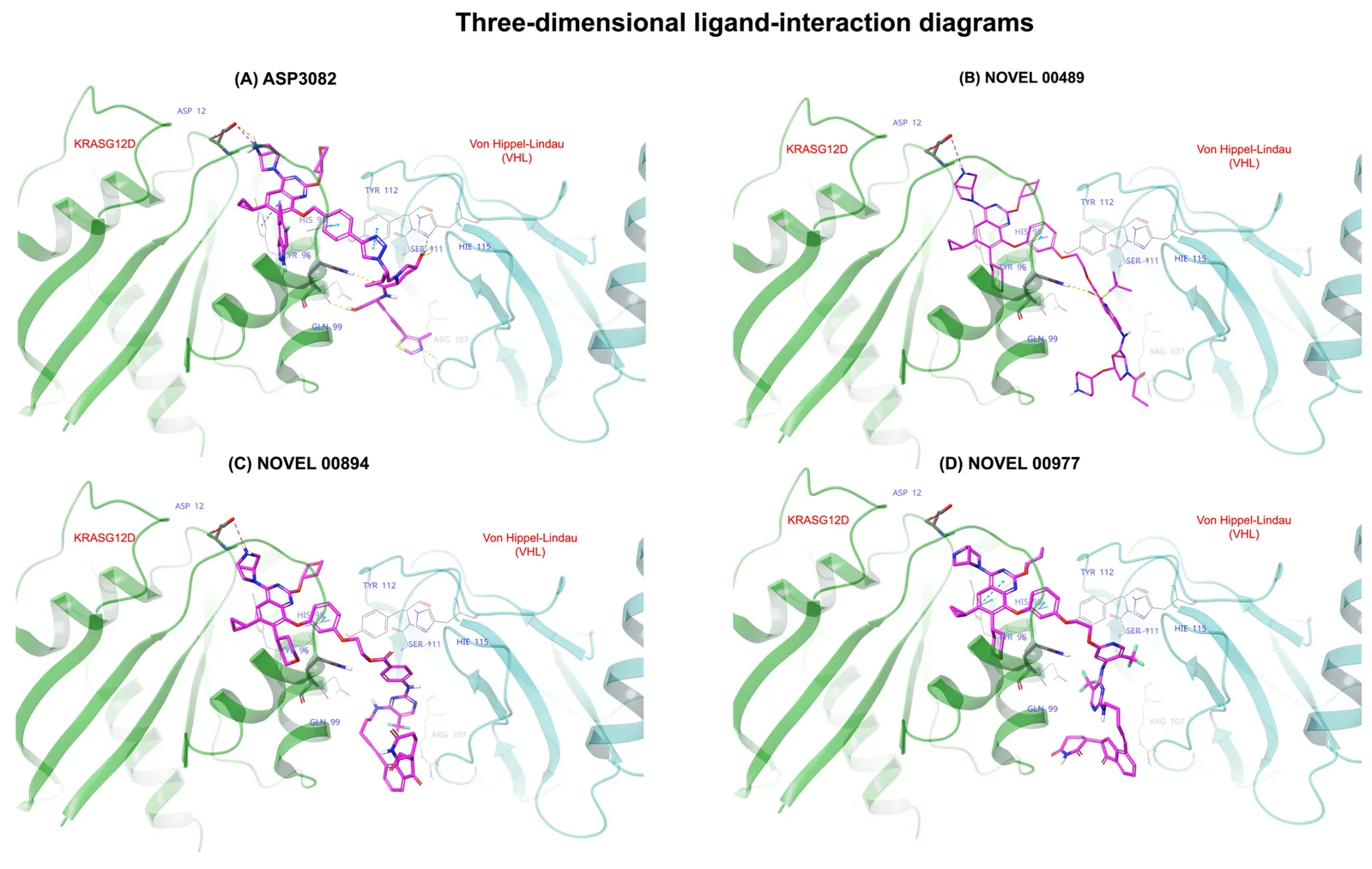
Three-dimensional ligand-interaction diagrams of the reference ASP-3082 and the top three novel KRASG12D PROTAC chimeras rendered from the top-scoring Glide SP/Prime MM-GBSA pose against PDB 9L6F. Green ribbons show KRASG12D, which hosts the ligand-binding Switch-II pocket, including the diagnostic Asp12 residue defined by the G12D mutation. Turquoise ribbons show VHL hosts the E3-recruiter binding pocket. (**A**) ASP3082 (positive control): canonical KRASG12D Asp12 salt bridge from the diazabicycloheptane-bridged amine, plus VHL hydroxyproline anchoring via hydrogen bonds to His115 and Tyr112. (**B**) NOVEL00489 (lead): conserved Asp12 salt bridge on KRASG12D AND a new second salt bridge from the piperidinyl protonated amine to Asp105 on VHL, plus a supplementary Arg102 π–cation interaction. (**C**) NOVEL00894 conserved Asp12 salt bridge; ASP-3082 hydroxyproline-VHL module replaced by a glutarimide CRBN-binding ligand that engages Pro97, illustrating the operationalization of the cluster-guided CRBN recommendation. (**D**) NOVEL00977 conserved Asp12 salt bridge; pyrimidine-CF_3_ spacer engages His110 through a hydrogen bond to the pyrimidine nitrogen, with the two trifluoromethyl groups occupying para/meta positions.

To investigate the structural features underlying the molecular mechanics generalized Born surface area (MM-GBSA) ranking, three-dimensional (3D) interaction maps were generated for ASP3082 and the three prioritized chimeras, NOVEL 00489, NOVEL 00894, and NOVEL 00977 (Figure 11). The maps were derived from the highest-ranked poses obtained by core-constrained Glide standard precision (SP) docking, followed by Prime MM-GBSA rescoring using Protein Data Bank (PDB) structure 9L6F [23]. This experimentally determined structure contains guanosine diphosphate-bound KRASG12D, ASP3082, and the von Hippel–Lindau (VHL)–Elongin B-Elongin C complex. Accordingly, interactions across both the KRASG12D and VHL surfaces were evaluated for ASP3082 and the VHL-recruiting NOVEL 00489.

NOVEL 00894 and NOVEL 00977 contain glutarimide-based cereblon (CRBN) recruiters rather than VHL recruiters. Their 9L6F-derived poses were therefore interpreted only in terms of KRASG12D recognition, preservation of the ligand orientation, and the direction of linker extension from the target-binding pocket. Because CRBN is not present in 9L6F, the current models cannot establish specific CRBN contacts or demonstrate formation of productive KRAS G12D-chimera-CRBN ternary complexes.

Such conclusions would require separate ternary-complex modeling using an experimentally determined CRBN-containing structure. The interaction maps were assessed for hydrogen bonds, salt bridges, π-cation contacts, π-π stacking, hydrophobic contacts, and solvent exposure.

The interaction map of ASP3082 (Figure 11A) reproduced the principal features of its crystallographic binding mode in 9L6F. On the KRASG12D face, the protonated bridged amine of the diazabicyclo [2.2.1] heptane group formed the expected ionic interaction with mutant Asp12. This contact anchors the target-binding moiety within the Switch II pocket and contributes to recognition of the G12D mutant. The cyclopropyl, tetrahydropyran, indazole, and quinazoline regions occupied the surrounding hydrophobic contours of the pocket, with contacts involving Val9, Ala11, Thr58, Ala59, Tyr96, and Ile100. The quinazoline-containing aromatic surface packed against Tyr96, while the fluorinated region formed a close van der Waals contact with Ile100. The latter interaction was therefore interpreted as a hydrophobic contact rather than a conventional halogen bond.

The VHL-directed portion of ASP3082 also retained the characteristic interaction pattern of the crystallographic ternary complex. KRAS Arg102 was positioned near the isopropyl region of the VHL ligand, while KRAS Glu98 was positioned near the ligand hydroxyl group at the protein–protein interface. A further interprotein contact was observed between KRAS Glu62 and VHL Arg69. Within the VHL-binding region, the triazole-containing portion of ASP3082 interacted with VHL His115 and engaged in aromatic stacking with VHL Tyr112. Together, the Asp12 ionic anchor, the VHL-binding interactions, and the complementary contacts between KRAS and VHL establish the structural reference against which the novel chimeras were assessed.

The predicted interaction map of NOVEL 00489 (Figure 11B) retained the intended KRASG12D-binding orientation of the ASP3082-derived ligand. The protonated bridged amine remained directed towards Asp12, preserving the principal ionic interaction associated with mutant recognition. The cyclopropyl-, tetrahydropyran-, and quinazoline-containing regions also occupied positions comparable with those observed for ASP3082 within the Switch II pocket. Because this pose was obtained through core-constrained docking, preservation of the ligand geometry should be regarded as the expected outcome of the design strategy rather than as independent validation of the binding mode.

The most important difference between NOVEL 00489 and ASP3082 was observed in the linker and VHL-facing regions. The piperidinyl–ethoxy–aryl–sulfonyl–tert–butyl architecture introduced a second protonatable amine in addition to the Asp12-binding bridged amine. In the prepared model, the protonated piperidine was oriented towards an acidic region of the VHL surface that included Asp105, creating a plausible additional ionic interaction. The central aromatic region was also positioned close to KRAS Arg102 at the ternary interface, permitting an additional π-cation contact. Meanwhile, the hydroxyproline-containing recruiter maintained an appropriate orientation within the VHL recognition pocket.

This extended electrostatic network offers a plausible structural explanation for the energetic profile of NOVEL 00489. Its MM-GBSA Coulombic contribution improved from −51.41 kcal per mole for ASP3082 to −74.37 kcal per mole, representing a change of approximately −23.0 kcal per mole. In contrast, the van der Waals and lipophilic contributions remained similar between the two compounds. The interaction map therefore suggests that the calculated 5.9 kcal per mole improvement in binding energy was driven mainly by the additional electrostatic contacts rather than by a nonspecific increase in hydrophobic burial. These contacts remain computational predictions, but the agreement between the interaction map and the energy decomposition provides a coherent structural basis for prioritizing NOVEL 00489.

The 9L6F-derived pose of NOVEL 00894 (Figure 11C) similarly preserved the intended KRASG12D-binding geometry. The bridged amine remained directed towards Asp12, the quinazoline-containing core occupied the Switch II pocket, and the surrounding hydrophobic substituents followed the general orientation observed for ASP3082. The distal linker and glutarimide-containing module extended away from the KRAS pocket through the solvent-exposed exit region without requiring a substantial displacement of the target-binding ligand. This finding supports the chemical compatibility of the CRBN-directed module with the conserved KRASG12D binding ligand.

However, the glutarimide group shown in Figure 11C was not docked into a CRBN-containing receptor. Contacts between the distal portion of NOVEL 00894 and residues belonging to the 9L6F KRAS-VHL system must therefore not be interpreted as CRBN-binding interactions. The present model demonstrates preservation of KRASG12D engagement and a feasible linker trajectory, but it does not establish the orientation or stability of the CRBN-recruiting end of the molecule. NOVEL 00894 should consequently be described as a chemical implementation of the cluster-guided CRBN recommendation rather than as a structurally validated KRASG12D-CRBN degrader.

The interaction map of NOVEL 00977 (Figure 11D) showed the same general preservation of the KRASG12D-binding mode. The protonated bridged amine remained positioned towards Asp12, while the aromatic moiety and hydrophobic substituents occupied the principal regions of the Switch II pocket. The rigid alkyne-containing linker and trifluoromethyl-substituted heteroaromatic spacer projected away from the KRAS-binding site and directed the distal glutarimide module towards the solvent-exposed E3 ubiquitin ligase-facing region. The two trifluoromethyl groups contributed additional lipophilic surface along the linker trajectory and were accommodated without an obvious steric disruption of the KRAS-bound ligand.

The current structural model, however, does not demonstrate CRBN engagement by NOVEL 00977. The predicted interactions of its distal glutarimide group with residues in 9L6F cannot be assigned to CRBN, as the receptor is absent from this structure. Similarly, the docking pose alone cannot explain its high predicted human oral absorption or confirm metabolic protection at particular cytochrome P450 oxidation sites. Those properties arise from the absorption, distribution, metabolism, excretion, and toxicity (ADMET) calculations discussed in the preceding section and require experimental pharmacokinetic and metabolic evaluation. The present interaction map supports only the preservation of the KRASG12D anchor and the steric feasibility of the linker exit direction.

Taken together, the structural analysis distinguishes two levels of evidence among the prioritized compounds. For NOVEL 00489, the VHL-compatible 9L6F model provides a complete predicted KRASG12D-chimera-VHL interaction network that is consistent with its favorable MM-GBSA decomposition. For NOVEL 00894 and NOVEL 00977, the same model supports retention of the mutant-directed KRAS-binding mode but cannot validate their CRBN-facing interactions. These results strengthen the prioritization of NOVEL 00489 as the leading VHL-recruiting candidate and identify dedicated CRBN ternary-complex modeling as the necessary next step for evaluating the biological potency of NOVEL 00894 for evaluating NOVEL 00894. 

## 3. Discussion

The central finding of this study is that multi-omics integration is substantially more informative when evaluated not only by its ability to reproduce known molecular subtypes but also by its capacity to generate stable, interpretable, and patient-resolved therapeutic hypotheses. Applying sixteen integration approaches to the same colon adenocarcinoma cohort under a unified scoring framework revealed striking differences in the structures they recovered. Similarity network fusion assigned 422 of 425 tumor profiles to a single dominant cluster while retaining an adjusted Rand index (ARI) of 0.32. Considered alone, this value might appear to indicate partial agreement with the reference classification. Viewed together with cluster balance, however, it exposes a partition with little practical value. Several deep generative methods showed the opposite pattern, achieving ARI values above 0.85 while producing silhouette widths below 0.30. Their labels broadly resembled the consensus molecular subtype (CMS) classification, but the corresponding latent spaces did not clearly separate the groups.

BLISS avoided both the cluster-collapse behavior observed for SNF and the weak internal separation produced by several deep generative methods. Its benchmark performance must, however, be interpreted in the context of its learning regime. BLISS uses PCA- and k-means-derived pseudo-labels to guide its triplet loss, whereas purely unsupervised comparator methods receive no analogous supervisory signal. The observed differences therefore reflect the complete BLISS framework, including pseudo-label-guided metric learning, and cannot be attributed exclusively to its network architecture. Within this context, BLISS achieved the highest silhouette width in the benchmark, at 0.78, while preserving balanced group sizes, a bootstrap stability of 0.93, and an ARI of 0.90 against the CMS classification. The importance of this result lies less in the superiority of a single numerical score than in the agreement among several complementary evaluation criteria. External label recovery, internal separation, prognostic association, cluster balance, bootstrap reproducibility, omics-layer dependence, and sample-level confidence describe different properties of an integration model, and no individual criterion can substitute for the others. The benchmark therefore supports a multi-objective evaluation framework in which the supervision regime is reported alongside performance, stability, and biological coherence.

Within the synthetic benchmark, the profile-specific top-ranked candidate differed from the corresponding cluster-level top-ranked candidate in 82.6% of tumor profiles. This comparison demonstrates that aggregation can alter the output of the computational scoring procedure. It does not indicate that a profile requires a different drug or that either ranking is clinically preferable because no treatment-response outcomes were available. The label-permutation analysis assesses concordance with the observed cluster assignments rather than treatment benefit. These rankings should therefore be regarded as hypothesis-generating outputs requiring experimental validation.

Most integration studies are designed to recover disease subtypes, associate them with prognosis, and describe their biological characteristics. Those are necessary achievements, but they do not by themselves produce an actionable decision. The clinically meaningful endpoint is not simply whether a tumor belongs to a recognized subtype, but whether the integrated molecular representation can support a transparent and testable therapeutic hypothesis for that tumor. The present framework, therefore, shifts the analytical focus from classification alone towards design. A related trajectory has been reported in head and neck squamous cell carcinoma, where AI-driven multi-omics analysis of cancer-associated fibroblasts identified heparin-binding epidermal growth factor-like growth factor as a prognostic marker and prioritized Epothilone B as a candidate compound [18]. The present work extends this principle by connecting tumor stratification to both drug prioritization and the design of new bifunctional degraders.

The principal novelty of the study is architectural rather than dependent on any single algorithm. BLISS, the drug-ranking module, the E3 ligase selection framework, and the shape-anchored chemical enumeration pipeline form a continuous sequence from molecular data to experimentally addressable structures. Previous studies have commonly stopped after identifying a subtype, pathway, biomarker, or repurposing candidate. Here, the output of integration is carried forward into a second biological decision, namely the choice of E3 ubiquitin ligase, and then into a third design decision, namely the construction of a proteolysis-targeting chimera (PROTAC) that implements the selected target and recruiter chemistry. The result is not merely a list of compounds associated with a cluster. It is a set of explicit molecular hypotheses in which the target-binding ligand, linker architecture, E3 recruiter, predicted interaction network, and physicochemical profile can all be examined independently.

KRASG12D was an appropriate target for demonstrating this progression. Mitogen-activated protein kinase (MAPK) signaling ranked among the leading pathways at the cohort level and was particularly prominent in the mesenchymal group. This biological signal converged with a rapidly advancing therapeutic landscape. ASP3082, now clinically known as setidegrasib, established that selective degradation of KRASG12D is structurally and pharmacologically feasible and has progressed into clinical development under NCT05382559 [23]. More recent clinical findings have also provided early evidence of antitumor activity in KRASG12D-mutant cancers. ACBI3 provides a complementary proof of principle for von Hippel–Lindau protein (VHL)-mediated degradation across multiple oncogenic KRAS variants, including G12D, although it is more accurately classified as a pan-KRAS degrader than as a G12D-specific degrader [24]. The frequency of KRASG12D across KRAS-mutant tumors and its substantial representation in colorectal cancer further strengthen its relevance as a translational test case.

The importance of integrated molecular context is greater for a degrader than for a conventional occupancy inhibitor. An inhibitor must encounter an accessible target in an appropriate cellular state. A PROTAC must additionally recruit a sufficiently expressed and functional E3 ligase, form a productive ternary complex, orient lysine residues for ubiquitination, enter the relevant cellular compartment, and maintain adequate exposure. Target abundance alone is therefore insufficient for rational degrader selection. E3 ligase expression, localization, ligandability, essentiality, tissue distribution, and compatibility with the target surface all contribute to the probability of productive degradation. Contemporary E3 ligase selection frameworks increasingly recognize these variables as central rather than secondary design considerations.

The cluster-specific E3 recommendations reflect this principle. Cereblon (CRBN) was prioritized for the canonical and metabolic groups, DDB1- and CUL4-associated factor 1 (DCAF1) for the immune group, and kelch domain-containing protein 2 (KLHDC2) for the mesenchymal group. None of the four groups selected VHL as the leading cluster-matched ligase. This result yields two distinct, scientifically useful prioritization routes. NOVEL 00489 is the strongest compound within the structure-based KRASG12D-VHL comparison, whereas NOVEL 00894 is more closely aligned with the CRBN recommendation derived from the canonical and metabolic clusters. These compounds should not be presented as competing answers to the same question. NOVEL 00489 represents optimization within a clinically validated VHL ternary-complex architecture. NOVEL 00894 represents an attempt to translate the omics-derived E3 preference into a different recruiter class. The distinction clarifies how molecular context and structural optimization can provide complementary rather than interchangeable design criteria. Recruiter transfer cannot be assumed to succeed for every target, and an experimental comparison remains necessary to determine whether the cluster-matched ligase yields more efficient degradation [45].

The shape-anchored enumeration pipeline provides the chemical bridge between these analytical levels. The validated KRAS-binding portion of ASP3082 was retained while variation was introduced principally within the linker and recruiter regions. This design preserves the experimentally established ligand exit geometry while allowing the remainder of the chimera to explore new chemical space. The subsequent core-constrained docking step therefore serves as a structural compatibility filter, identifying candidates that accommodate linker and recruiter diversity without requiring a major reorientation of the KRAS-binding core.

A second practical advance was the use of a two-stage similarity cascade. Alignment-free two-dimensional screening with Morgan fingerprints reduced the initial search space, after which the remaining structures were evaluated using ultrafast shape recognition with CREDO atom types (USRCAT) across three-dimensional conformer ensembles. Morgan fingerprints and shape-based screening are individually established approaches, and Morgan representations have demonstrated value in virtual ligand screening. Their sequential application was particularly effective for the present ligand-anchored library, reducing an initially impractical calculation to approximately 30 to 60 min for 10,000 candidates on the available workstation. The methodological innovation lies in how these components were assembled into a reproducible workflow that moves from a reference ternary structure to a ranked structure-data file (SDF) containing chemically diverse, ligand-preserving candidates.

The three prioritized compounds illustrate the different design opportunities captured by this workflow. NOVEL 00489 represents electrostatic optimization within a VHL-recruiting architecture. NOVEL 00894 replaces the VHL-directed recruiter with a glutarimide-containing CRBN module, translating the cluster-guided E3 hypothesis into a tangible chemical design. NOVEL 00977 explores a rigid, trifluoromethyl-substituted linker with a markedly different predicted property profile. The value of the resulting series is therefore not confined to identifying a single highest-ranked molecule. It provides three experimentally distinguishable strategies within the same KRAS-binding ligand family: strengthening the predicted ternary interface, changing the recruited ligase, and altering linker rigidity and physicochemical behavior.

NOVEL 00489 emerged as the strongest overall candidate in the KRASG12D-VHL structural comparison. Its Prime molecular mechanics generalized Born surface area (MM-GBSA) score was 5.9 kcal mol^−1^ more favorable than that of ASP3082. Energy decomposition attributed the largest difference to the Coulombic term, which improved by approximately 23 kcal mol^−1^, whereas the van der Waals and lipophilic contributions remained broadly comparable. This pattern indicates that the ranking was not driven solely by the increase in hydrophobic surface area. Instead, it points towards a more favorable electrostatic arrangement introduced by the piperidinyl–ethoxy–aryl–sulfonyl–tert–butyl linker.

The absorption, distribution, metabolism, excretion, and toxicity (ADMET) calculations reinforce the prioritization of NOVEL 00489 at the comparative level. Relative to ASP3082, the compound showed lower calculated lipophilicity, reduced polar surface area, a modestly higher predicted percentage human absorption, and lower predicted human serum albumin binding. These changes are meaningful because they suggest that the improved interaction score was not achieved solely by increasing lipophilicity or molecular size. At the same time, the high molecular weight, large polar surface area, and numerous QikProp property deviations remain characteristic challenges for bifunctional degraders. NOVEL 00489 is therefore best described as the most balanced computational candidate in the present series rather than as a conventionally drug-like molecule.

The 3D interaction analysis provides a structural explanation for its energetic profile. Protein Data Bank (PDB) structure 9L6F contains the experimentally determined KRASG12D-ASP3082-VHL-Elongin B-Elongin C ternary complex and is therefore directly appropriate for analyzing ASP3082 and the VHL-directed NOVEL 00489 [43,44]. In the constrained pose of NOVEL 00489, the protonated bridged amine retained its ionic interaction with mutant Asp12, while the additional protonated piperidine was positioned near VHL Asp105. The central aromatic region also approached KRAS Arg102, creating a plausible supplementary π-cation interaction. Together, these contacts define a dual-anchor hypothesis in which the conserved KRAS-facing ionic interaction is complemented by a second electrostatic contact on the VHL-facing side.

This interpretation is consistent with the MM-GBSA decomposition, but its importance lies in its testability through experiments. The model predicts that reducing the piperidine’s basicity or replacing it with a geometrically similar neutral group should weaken the calculated Coulombic advantage. N-methylation would not provide a suitable control because a tertiary piperidine remains protonatable. More informative matched pairs would include a lactam analog, an oxygen-containing ring, or another neutral isostere that preserves the approximate linker dimensions while disrupting the proposed Asp105 interaction. Comparison of these analogs by ternary-complex binding, cooperativity, and cellular degradation would directly test whether the predicted second ionic anchor contributes to activity [45]. Such matched molecular pair analysis would convert the docking interpretation into a falsifiable medicinal chemistry hypothesis [46].

The preservation of the Asp12 interaction across the three prioritized structures also demonstrates that the enumeration pipeline retained the intended KRAS-binding geometry. This outcome should be interpreted in the context of the core constraint, which restricted the RMSD of the conserved ligand relative to the crystallographic pose. The result therefore establishes structural consistency with the design rule rather than providing independent proof of binding or selectivity. Even so, this consistency is important. The purpose of ligand-anchored PROTAC design is to maintain a validated target-binding orientation while allowing chemical divergence in the linker and E3 recruiter regions [47,48]. The present workflow achieves that objective without forcing the complete chimera to remain globally similar to ASP3082.

NOVEL 00894 provides the most direct chemical implementation of the CRBN recommendation. Its conserved ligand retained the intended KRASG12D orientation, while the linker projected the glutarimide-containing recruiter towards the solvent-exposed region of the complex. Glutarimide motifs are well established in thalidomide-derived CRBN ligands and in CRBN-recruiting PROTACs [19,20]. The present 9L6F model, however, contains VHL rather than CRBN. It can therefore establish the linker’s compatibility with the KRAS-bound ligand, but it cannot define the final CRBN-binding orientation or identify authentic CRBN contacts. The significance of NOVEL 00894 is, consequently, both chemical and strategic: it shows that the cluster-guided CRBN hypothesis can be expressed as a synthetically recognizable KRASG12D chimera without disrupting the constrained target-binding geometry.

NOVEL 00977 occupies a different region of the design landscape. Its two trifluoromethyl groups and rigid heteroaromatic linker resulted in a predicted human absorption value of 85.9%, but were accompanied by very high calculated lipophilicity and extremely low aqueous solubility. The compound therefore demonstrates a pronounced physicochemical trade-off. Trifluoromethyl substitution can alter conformation, membrane partitioning, metabolic susceptibility, and local binding interactions [44], but the docking pose alone cannot establish that the substituted positions are protected from cytochrome P450 oxidation or that the fluorinated linker is responsible for the predicted absorption value. Rather than treating NOVEL 00977 as an orally optimized candidate, it is more informative to view it as a source of linker features that can be dissected individually. Retaining one fluorinated substituent, reducing total aromatic surface area, or combining selected rigidity elements with a less lipophilic scaffold may provide a more productive route than transferring the complete linker into NOVEL 00489.

Taken together, the interaction maps transform the computational ranking into a series of specific mechanistic questions. For NOVEL 00489, the key question is whether the predicted Asp105-facing interaction increases ternary-complex stability, cooperativity, or degradation efficiency. For NOVEL 00894, the question is whether CRBN can form a productive ternary complex with the conserved KRASG12D binding ligand and the proposed linker geometry. For NOVEL 00977, the question is which linker features, if any, improve permeability or metabolic stability without reproducing its severe lipophilicity and solubility liabilities. The value of the structural analysis is therefore not that it proves the compounds will function as predicted, but that it identifies the molecular features that should be altered, retained, or challenged experimentally.

The most direct validation path begins with NOVEL 00489. The compound should be synthesized and compared head-to-head with ASP3082 in KRASG12D-mutant cell lines using established ASP3082 protocols [23]. The comparison should include target engagement, ternary complex formation, KRAS ubiquitination, proteasome dependence, the concentration required for 50% degradation (DC50), maximum degradation (Dmax), the duration of degradation after washout, and proteome-wide selectivity. A neutral analog of the piperidine-containing linker would provide a mechanistic control for the proposed Asp105 interaction. Biophysical measurements of binary and ternary affinity would determine whether the favorable computational ranking is accompanied by increased complex stability or cooperativity.

A second experimental series should address the cluster-guided E3 hypothesis. PROTACs that share the same ASP3082-derived KRAS-binding ligand but recruit CRBN, DCAF1, KLHDC2, or VHL could be tested in molecularly characterized colon adenocarcinoma models assigned to the corresponding BLISS groups. Patient-derived organoids would be particularly informative because they preserve intertumoral heterogeneity more effectively than a single cell-line panel. The central prediction is not simply that one ligase will universally outperform the others. The relative efficiency and durability of degradation will vary with the molecular and E3 ligase context identified by the integrated omics model. A positive result would establish a direct connection between tumor stratification and degrader architecture. A negative result would be equally informative, as it would identify which molecular measurements are insufficient to predict productive ligase recruitment.

The broader implication of this work is that precision oncology should not treat molecular integration, therapeutic prioritization, and compound design as isolated analytical tasks. Their separation creates an avoidable gap between biological interpretation and experimental action. BLISS provides a stable representation of tumor heterogeneity, but its greater value emerges when that representation is used to expose patient-level therapeutic differences, nominate contextually relevant E3 ligases, and constrain compound design to test those predictions. In this sense, the framework advances from describing tumor states to engineering interventions around them.

The contribution of the study is therefore not a claim that one computational score has already identified a clinically superior degrader. Its contribution is the creation of a reproducible route from heterogeneous molecular data to explicit, synthesis-ready, and mechanistically testable chemical hypotheses. BLISS defines the disease context, the E3 module introduces the degradation context, and the shape-anchored pipeline converts both into candidate structures. As precision oncology expands beyond occupancy-based inhibitors towards catalytic degraders, antibody-drug conjugates with selectable payloads, and messenger ribonucleic acid vaccines with selectable antigens, the central challenge will increasingly be one of design rather than classification. Multi-omics integration will be most valuable when it does not end with a subtype label, but continues until the biological distinction has been translated into an intervention that can be built, tested, and refined.

The contrast between the synthetic benchmark and the real cohorts is informative. In real tumors, unsupervised multi-omics integration recovers an expression-defined subtype taxonomy only modestly, and adding non-expression layers neither improves nor dilutes agreement with CMS. This tempers expectations for unsupervised integration as a subtype-recovery tool and argues for supervised or expression-anchored strategies when the target taxonomy is expression-defined. The consistency of the survival finding across synthetic and real data, in which stage dominates outcome while molecular clusters do not, strengthens confidence in that specific conclusion.

The discovery cohort is synthetic and reproduces marginal distributions but not full cross-omic covariance, batch structure, missingness, or tumor heterogeneity; it therefore serves only as a controlled benchmark. The real cohort is limited to 186 complete-case tumors, CPTAC follow-up is short, and the proposed BLISS model is weakly supervised rather than fully unsupervised. The drug and degrader analyses are computational: target expression is not drug sensitivity, binary docking does not establish a productive ternary complex, and MM GBSA is a binding estimate rather than a measure of degradation. No synthesis, binding, ternary-complex, degradation, or cell-based assays were performed. All compounds are candidate hypotheses, and their synthesis and experimental validation are planned for future work after lead optimization.

## 4. Materials and Methods

### 4.1. Cohort Construction

The cohort comprises 425 primary tumors and 41 matched adjacent simulated normal profiles (total *n* = 466). Per-sample features were generated by sampling from distributions parameterized against published references. The five molecular layers were mRNA expression (3000 genes, log-normal distribution), DNA methylation (1500 CpG probes, beta distribution), CNV (2500 segments, Gaussian around integer copy states), miRNA (300 mature species, negative binomial distribution), and RPPA (200 antibodies, Gaussian distribution). Driver mutation calls for APC, KRAS, B-Raf proto-oncogene serine/threonine kinase (BRAF), TP53, PIK3CA, mothers against decapentaplegic homolog 4 (SMAD4), and F-box and WD repeat domain-containing 7 (FBXW7) were sampled at frequencies reported in Bailey 2018 [49]. CMS labels were assigned at the Guinney 2015 proportions [4]. MSI status, age at diagnosis (continuous, median 67 years, interquartile range 60 to 75), AJCC stage (I to IV at TCGA proportions), and censored overall survival follow-up were generated to match marginal TCGA distributions [36]. The random seed (20251124) is fixed in the code so that all results are reproducible.

### 4.2. Preprocessing

Each omics layer was z-score standardized for each feature in the tumor cohort. The top 2000 most variable mRNA genes, the top 1000 most variable methylation probes, the top 1000 most variable CNV segments, all 300 miRNAs, and all 200 RPPA proteins were retained for integration. No batch correction was applied because the cohort is internally consistent by construction.

### 4.3. Integration Methods Benchmarked

Sixteen multi-omics integration methods spanning four families were benchmarked. The classical statistical family included principal component analysis on concatenated layers, joint and individual variation explained (JIVE), and a MOFA-inspired factorization [7,8,9]. The similarity-based family included SNF and neighborhood embedding multi-omics (NEMO) [10,50]. The autoencoder family included a linear masked autoencoder and a deep masked autoencoder. The deep generative family included DeepDRA, Dr.VAE, a multi-omics integrative conditional variational autoencoder, a multi-omics visible drug activity prediction, a multi-omics factor graph convolutional network, a tumor subtype graph convolutional neural network, non-negative dimensionality reduction by sparse principal components, multi-omics late integration, and a Super.FELT-inspired supervised feature-extraction formulation [14,15,16,17,30]. Each method produced a 16-dimensional sample-level embedding, from which four clusters were obtained by spectral clustering on the cosine-similarity graph. The MOFA-inspired and Super.FELT-inspired models were custom, benchmark-compatible formulations rather than executions of the official codebases, and no formal equivalence testing was performed. Accordingly, these formulations should not be interpreted as exact reproductions of the published methods or as estimates of the performance of their official implementations. The benchmark included methods operating under different learning regimes. BLISS was classified as pseudo-label-guided and weakly supervised because its triplet loss used cluster-derived pseudo-labels, whereas purely unsupervised comparator methods received no corresponding supervisory signal. Performance rankings were therefore interpreted as comparisons of complete integration frameworks under common preprocessing and scoring procedures, rather than as comparisons among methods trained under identical supervision conditions.

### 4.4. BLISS Architecture

BLISS combines three components. Separate linear encoders map each omics layer into a shared 16-dimensional latent space. The layer-specific embeddings are concatenated, passed through layer normalization and a GELU nonlinearity, and projected into a 16-dimensional L2-normalized joint representation. Three loss terms drive training. Before neural-network training, pseudo-labels were generated by applying PCA to the concatenated omics layers, followed by k-means clustering with k = 4. These cluster assignments were used to construct the triplets, with the anchor and positive samples drawn from the same pseudo-cluster and the negative sample drawn from a different pseudo-cluster. The triplet metric loss (λ_triplet = 1.0), inspired by the triplet-loss principle used in Super.FELT [30], pulls samples assigned to the same pseudo-cluster closer in the latent space while separating samples assigned to different pseudo-clusters. No clinical, survival, CMS, treatment response, or other outcome labels were used to generate the pseudo-labels or to train BLISS. Nevertheless, because these data-derived pseudo-labels directly guide representation learning, BLISS is classified as weakly supervised rather than fully unsupervised.

The factor reconstruction loss (λ_factor = 0.3), adapted from the shared-factor reconstruction principle of MOFA [9], reconstructs each input layer from the shared latent representation. These loss components were implemented specifically for BLISS and do not reproduce the complete Super.FELT or MOFA algorithms. The balance regularizer (λ_balance = 0.7), introduced in this work, is the KL divergence between the marginal cluster distribution and a uniform distribution over four clusters. Because λ_balance directly encourages uniform cluster frequencies, cluster balance is not an independent evaluation criterion for BLISS and was therefore interpreted with caution. Training was performed for 200 epochs using a single GPU.

### 4.5. Scoring, Cluster Balance, and Three New Evaluation Axes

Each partition was scored on four primary axes. ARI and normalized mutual information were computed against the CMS labels. Silhouette width was computed on the cosine distance of the integrated embedding. Multivariate log-rank chi-square was computed on overall survival across the four clusters. Cluster balance was defined as the size of the smallest cluster divided by the size of the largest, with a minimum floor of 5 percent below which a partition was marked as degenerate. Because BLISS explicitly optimizes a related quantity via the λ_balance term, its cluster-balance score was interpreted with caution and not as independent evidence of comparative superiority. A composite rank with a penalty for degenerate partitions was used to nominate the winning method. The three new evaluation axes were repeated-subsampling stability, calculated as the mean pairwise adjusted Rand index across fifteen independently drawn 80% patient subsamples for each method, layer ablation (the ARI drop when each layer was removed individually), and per-patient confidence (the fraction of bootstrap iterations in which a patient was assigned to its modal cluster). This analysis was used as an exploratory sensitivity diagnostic. Because only fifteen subsamples were evaluated, no formal uncertainty interval was estimated, and the result was not interpreted as a precise measure of long-run stability.

### 4.6. Differential Expression, Pathway Enrichment, and Classifier Positive Control

Tumor versus normal differential expression was computed with a two-sample *t*-test per gene with Benjamini–Hochberg false discovery rate control at α = 0.05 [51]. Pathway overrepresentation analysis was performed against the Molecular Signatures Database hallmark gene sets [29] using a Fisher exact test. Three classifiers (L2-regularized logistic regression, random forest, extreme gradient boosting) were trained to discriminate tumor from simulated normal profiles in the integrated feature space and evaluated using five-fold stratified cross-validation as a positive control.

### 4.7. Survival Analysis

Kaplan–Meier curves were estimated for each integrated cluster and each AJCC stage [37]. Multivariate log-rank chi-square was computed across groups. A Cox proportional hazards model included age (per decade), stage (III/IV versus I/II), MSI status, and integrated cluster as covariates [52].

### 4.8. Real Cohort Acquisition and Independent Subtype Labeling

Real TCGA COAD data (five layers with clinical and survival information) were obtained from the UCSC Xena platform [53] and harmonized into a complete-case cohort of 186 tumors. CMS labels were derived independently from real expression data using CMScaller (v0.99.2) [54] and were used only for post hoc evaluation, never for training. The independent CPTAC 2019 prospective cohort [31] provided external validation using the shared expression, copy number, and miRNA layers. Each layer was reduced to its most variable features before integration.

### 4.9. Integration Evaluation, Stability, and Null Model

Each layer was standardized and concatenated; principal component analysis, followed by k-means (k = 4), produced clusters, which were evaluated using the Adjusted Rand Index and silhouette width against the independent labels. A permutation null model (10,000 permutations) was used to assess per-sample drug divergence.

### 4.10. Pharmacogenomic Validation

Computational drug rankings were validated against the public PRISM viability screen [47] restricted to colorectal cell lines. The primary drug score used only molecular evidence, with curated subtype annotation retained solely for a labeled sensitivity analysis.

### 4.11. Drug and E3 Ubiquitin Ligase Prioritization

A curated panel of 39 drugs with annotated gene targets was scored per cluster. The per-cluster drug score was the product of (i) cluster mean expression of the drug target normalized against the cohort, (ii) prevalence of the drug target above the median in the cluster, and (iii) a curated annotation weight that captures whether the drug is clinically relevant for the subtype biology. The panel spans CDK4/6 inhibitors (palbociclib, ribociclib, abemaciclib), immune checkpoint blockade (pembrolizumab, nivolumab, ipilimumab, atezolizumab, cemiplimab), MAPK axis inhibitors (trametinib, cobimetinib, encorafenib, dabrafenib), PI3K, AKT, and mTOR inhibitors (alpelisib, capivasertib, everolimus, sirolimus, temsirolimus), the Wnt-directed agent vantictumab, and cytotoxics (oxaliplatin, irinotecan, fluorouracil). Patient-specific drug ranking replaced the cluster mean with each patient’s own target expression vector.

The E3 ubiquitin ligase analysis was expression-driven. Relative messenger RNA expression of CRBN, DCAF1, KLHDC2, and VHL was summarized within the BLISS clusters to nominate cluster-associated recruiter hypotheses. Recruiter availability was considered separately during chemical enumeration, which admitted CRBN- and VHL-binding pharmacophores. The expression analysis did not assess protein abundance, subcellular localization, target-ligase colocalization, ligase activity, ternary-complex cooperativity, lysine presentation, ubiquitination, or degradation competence. Accordingly, the E3 assignments were interpreted as hypothesis-generating only.

### 4.12. De Novo PROTAC Design: Computational Resources and Software

All computational work was performed on a workstation equipped with an Intel Xeon Gold 6130 central processing unit (CPU; 32 cores at 2.10 GHz), 192 GB of system memory, and an NVIDIA Quadro P4000 GPU (8 GB video RAM, Compute Unified Device Architecture (CUDA) 12.2) running Ubuntu Linux. The cheminformatics pipeline was implemented in Python 3.9 within a dedicated Conda environment, with RDKit (release 2022.09) serving as the principal toolkit for molecular handling, fragmentation, and 3D shape analysis. Structure preparation, conformer generation, molecular docking, free-energy estimation, and ADMET profiling were performed using the Schrödinger Suite (release 2025 1; Schrödinger, LLC, New York, NY, USA).

### 4.13. Reference Data and Target Structure

The clinical KRASG12D selective degrader ASP 3082 (setidegrasib, CAS 2821793 99 9; degradation potency DC50 = 38 nM) was selected as the structural anchor for the entire design campaign on the basis of its validated co-crystal structure with KRASG12D · GDP in complex with the VCB heterotrimer (PDB entry 9L6F, 3.18 Å resolution) [23]. The bound conformation of ASP 3082 was extracted from 9L6F as a Tripos MOL2 file (146 atoms) and used both as a 3D shape query and as the source of the conserved ligand. Two additional KRASG12D degraders, RP03707 (DC50 = 5 nM) [48], and ZJK 807 (DC50 = 79.5 nM) [55], together with a 17-compound in-house KRASG12D PROTAC set, were retained as orthogonal references for assessing chemical novelty. A general PROTAC chemistry library of 9381 fully annotated compounds was assembled from PROTAC DB 3.0 [45] covering 569 ligands, 2753 linkers, 107 E3 ligands, and the associated degradation data, target identifiers, and physicochemical descriptors.

### 4.14. Decomposition of the Reference and Source Library

The ASP 3082 reference and every entry of the 9381-compound PROTAC DB library were fragmented using the BRICS algorithm [34] as implemented in RDKit (rdkit. Chem.BRICS. BRICS Decompose, minimum fragment size 5 heavy atoms for the library and 8 heavy atoms for ASP 3082). BRICS preserves chemically meaningful retrosynthetic exit vectors, encoded as numbered dummy atoms, that allow downstream fragment reconnection without violating valence rules. Fragments were automatically classified into three pools based on substructure pattern matching: (i) E3 ligase recruiters, defined as fragments matching the CRBN binding glutarimide pharmacophore (SMARTS O=C1NC(=O)CCC1) or the VHL binding hydroxyproline scaffold (SMARTS C1C(O)CC(C(=O)N)N1), restricted to heavy atom counts between 10 and 45; (ii) linkers, defined as fragments lacking either E3 binding pharmacophore, with heavy atom counts between 4 and 20 [47]; and (iii) the KRASG12D binding ligand of ASP 3082, identified as the largest BRICS fragment lacking either E3 pharmacophore, comprising the 2 (tetrahydropyran 4 yloxy) quinazoline core decorated with a 6 cyclopropyl group, a 4 (2,5 diazabicyclo [2.2.1]heptan 2 yl) bridged amine (the key Asp12 contact, observed in 9L6F at 2.78 Å), a 7 (6 fluoro 5 methyl 1H indazol 4 yl) substituent, and a methylene aryl exit vector for linker attachment. Following deduplication, the resulting building block pools contained 3179 unique linkers, 1 unique E3 recruiter retained after the strict substructure filter, and an additional set of 500 chemically diverse fragments admitted from the library to preserve combinatorial breadth.

### 4.15. Anchored Combinatorial Enumeration

Novel PROTAC candidates were assembled using Python 3.9RDKit’s BRICS Build algorithm, with the ASP 3082 KRAS binding ligand specified as a mandatory seed fragment, ensuring that every output molecule retains the validated KRASG12D binding chemotype. The builder was executed with only Complete Mols = True (rejecting partially assembled intermediates), scramble Reagents = True (randomizing fragment selection at each step), and maxDepth = 4 (limiting the assembly tree depth). Single component connectivity was enforced after each assembly step by requiring len(Chem. GetMolFrags(mol)) == 1, eliminating the disconnected fragment artifact characteristic of 3D diffusion-based generators [32,33]. Candidates were further constrained to molecular weights between 700 and 1500 Da; the presence of either a CRBN- or VHL-binding pharmacophore; and non-identity to any entry in PROTAC DB 3.0 or the reference set, as established by canonical SMILES comparison. A maximum of 15,000 candidates was enumerated to allow tractable downstream scoring.

### 4.16. Two-Stage 3D Shape Filtering

To bias the library toward analogs that occupy the same volume as ASP 3082 in the KRASG12D Switch II pocket while permitting linker and recruiter diversification, we applied a two-stage shape similarity filter. Stage 1 (two-dimensional pre-filter): each candidate was encoded as a Morgan circular fingerprint (radius 2, 2048-bit folded vector; equivalent to Extended Connectivity Fingerprint of diameter 4, ECFP4) using Python 3.9 RDKit.Chem.AllChem.GetMorganFingerprintAsBitVect, and the Tanimoto coefficient versus the ASP 3082 fingerprint was computed. Candidates with Tanimoto ≥ 0.20 were retained. Stage 2 (3D USRCAT shape similarity): survivors of Stage 1 were embedded in three dimensions using the ETKDGv3 distance geometry algorithm [37] with five conformers per molecule and MMFF94s force field optimization. The USRCAT shape descriptor [51], which augments the four classical Ultrafast Shape Recognition distance moments with twelve additional pharmacophore-typed moments (hydrophobic, aromatic, donor, acceptor) for a sixty-dimensional shape and feature signature, was computed for each conformer (rdkit.Chem.rdMolDescriptors.GetUSRCAT) and compared with the USRCAT vector of the ASP 3082-bound conformer using the inverse Manhattan similarity metric (GetUSRScore, range 0 to 1). Each molecule was assigned the maximum score across its five conformers, and only molecules with USRCAT score ≥ 0.15 were retained—a threshold calibrated for the high inherent flexibility of PROTAC-sized molecules (approximately 1100 Da).

### 4.17. Library Scoring and Selection

A reference-compatibility score was assigned to each shape-passing candidate by linearly combining: (i) the USRCAT shape score against ASP3082 (weight 100); (ii) the two-dimensional Morgan Tanimoto similarity to ASP3082 (weight 50); (iii) a bonus for molecular weight within 1000 to 1250 Da; (iv) a 9L6F-template-compatibility term for retention of the VHL recruiter used by ASP3082; and (v) a drug-likeness term favoring calculated octanol/water partition coefficients between 2 and 6. Candidates were ranked in descending order. The VHL-specific term reflects compatibility with the experimental structural template rather than an omics-derived preference for VHL. Because this term is structurally biased toward VHL-recruiting molecules, the score was not interpreted as an unbiased comparison between recruiter classes. The selected candidates were therefore discussed in terms of two distinct design routes: a VHL-compatible structure-optimization route and a CRBN-directed, cluster-aligned route.

### 4.18. Ligand Preparation, Conformer Generation, and Shape Screening

The 10,000-compound shape-anchored library was imported into Maestro and processed with LigPrep (Schrödinger Suite 2025 1) using the OPLS4 force field; ionization and tautomeric states were enumerated with Epik at pH 7.0 ± 2.0, stereoisomers were retained at the specified chirality, and a single low-energy ring conformation was generated per stereoisomer. For each prepared ligand, a bioactive conformer ensemble (up to 256 conformers per molecule, an energy window of 12.5 kcal/mol) was generated with ConfGen in rapid-intensive mode. The resulting Phase 3D database was screened with Phase Shape Screening (GPU-accelerated mode) against the bound conformation of ASP 3082 extracted from PDB 9L6F. Atom typing was set to pharmacophore features [39] with shape and feature contributions equally weighted, and the per-conformer Gaussian volume overlap Tanimoto coefficient was retained for downstream filtering.

### 4.19. Protein Preparation and Receptor Grid Generation

For protein preparation, the X ray coordinates of KRASG12D GDP in complex with ASP 3082 and the VCB complex (PDB 9L6F) were retrieved and processed with the Protein Preparation Wizard, Schrödinger Release 2025-1: bond orders were assigned, hydrogens added, missing side chain atoms rebuilt with Prime, protonation states predicted by PROPKA at pH 7.4, water molecules beyond 5 Å of the bound ligand removed, and the complex restrained minimized with the OPLS4 force field until heavy atom RMSD reached 0.30 Å. A 30 Å × 30 Å × 30 Å receptor grid was centered on the centroid of the bound ASP 3082, with a 14 Å inner box and a 20 Å outer box, using Receptor Grid Generation (Glide 9.6) [56].

### 4.20. Standard Precision Molecular Docking and Free Energy Estimation

Shape-passing ligands were docked into the prepared 9L6F grid using Glide SP, sampling flexible ring conformations, generating up to 25 poses per ligand, and writing the single best pose for each ligand for analysis. Core constrained docking was applied: the quinazoline 2 yl tetrahydropyran ligand atoms of the docked ligand were tethered to the corresponding atoms of the 9L6F bound conformation with a 0.5 Å RMSD tolerance, ensuring that the Asp12 contacting bridged amine of every analog is forced into the experimentally observed orientation, in line with the validated practice of core-constrained docking for congeneric series [52]. Binding free energies were estimated for the top-scoring poses using Prime MM GBSA with the VSGB 2.0 solvent model and the OPLS_2005 force field, allowing all residues within 5 Å of the ligand to relax during minimization. The MM GBSA estimator decomposes the binding free energy ΔG_bind into Coulomb, covalent, hydrogen bond, lipophilic, π–π packing, self-contact, generalized Born solvation, and van der Waals contributions, providing a chemically interpretable readout [57].

### 4.21. Drug Likeness and ADMET Profiling

Top ranked compounds were profiled with QikProp (Schrödinger Suite 2025 1) to predict 51 physicochemical and ADMET descriptors, including (i) Lipinski rule of five violations; (ii) Jorgensen rule of three violations; (iii) predicted octanol/water partition coefficient (QPlogPo/w); (iv) predicted aqueous solubility (QPlogS, mol per litre); (v) predicted Caco 2 cell permeability (QPPCaco, nm per second) and MDCK permeability; (vi) predicted hERG channel inhibition (QPlogHERG, μM); (vii) predicted human serum albumin binding (QPlogKhsa); (viii) predicted percent human oral absorption; (ix) topological polar surface area (PSA); and (x) the number of likely cytochrome P450 metabolic sites.

### 4.22. Software and Reproducibility

All analyses are implemented in Python 3.12 with PyTorch (v2.13.0) [58], scikit-learn (v1.9.0) [59], XGBoost (v3.4.1) [60], and lifelines (v0.30.3) [61] as core dependencies for the multi-omics pipeline, and Python 3.9, RDKit, and the Schrödinger Suite for the de novo PROTAC pipeline.

## 5. Conclusions

This study presents an integrated artificial intelligence-driven framework that connects multi-omics stratification with sample-resolved therapeutic prioritization, E3 ubiquitin ligase selection, and structure-guided proteolysis-targeting chimera design in colon adenocarcinoma. Balanced Latent Integration with Stability Selection achieved the highest silhouette width among the evaluated methods (0.78), while maintaining an adjusted Rand index of 0.90, balanced clusters, and a mean pairwise adjusted Rand index of 0.93 in an exploratory fifteen-subsample stability analysis. The finding that 85% of the simulated tumor profiles received a different top-ranked drug from their corresponding cluster average further demonstrates that cluster-level summaries can conceal molecular variation relevant to therapeutic prioritization.

The cluster-resolved analysis favored cereblon, DDB1- and CUL4-associated factor 1, or kelch domain-containing protein 2 over von Hippel–Lindau protein, depending on molecular context. The subsequent shape-anchored design workflow translated these biological hypotheses into chemically explicit KRASG12D degrader candidates. NOVEL 00894 provided a cereblon-directed chimera aligned with the canonical and metabolic cluster recommendations, whereas NOVEL 00489 emerged as the strongest candidate within the von Hippel–Lindau-compatible structural model. NOVEL 00489 produced a molecular mechanics generalized Born surface area score 5.9 kcal mol^−1^ more favorable than that of ASP3082, accompanied by an approximately 23 kcal mol^−1^ improvement in the Coulombic component and modest improvements in selected calculated physicochemical descriptors. Its predicted interaction network suggests a testable electrostatic contribution involving KRAS Asp12 and von Hippel–Lindau Asp105. All docking scores and MM-GBSA values represent binary binding estimates at the KRASG12D switch-II pocket and do not model the ternary KRASG12D: PROTAC: E3 complex. These PROTAC candidates are identified through computational prioritization and represent preliminary selections. To advance this research, synthesis and comprehensive biological experimentation are essential for subsequent optimization.

The principal advance of the framework lies in linking molecular stratification to synthesis-ready degrader hypotheses rather than ending with subtype classification or drug ranking. The resulting candidates provide defined experimental starting points for testing whether tumor-specific E3 ligase context can guide the selection and optimization of targeted protein degraders. This work therefore establishes a reproducible computational route from multi-omics heterogeneity to mechanistically interpretable and experimentally testable therapeutic designs for precision oncology.

## Data Availability

All data generated in this study are available within the article and its Supplementary Materials. The analysis code and additional generated datasets are available in the following GitHub repository https://github.com/KHALED-SULIMAN/AI-driven-multi-omics-integration-of-synthetic-colon-adenocar-cinoma-cohort (accessed on 6 July 2026).

## Supplementary Materials

The following supporting information can be downloaded at https://www.mdpi.com/article/10.3390/ijms27177511/s1.

## Abbreviations

The following abbreviations are used in this manuscript:

accptHB	QikProp predicted hydrogen-bond acceptor count
ADMET	absorption, distribution, metabolism, excretion, toxicity
AI	artificial intelligence
AJCC	American Joint Committee on Cancer
APC	adenomatous polyposis coli
AUC	area under the ROC curve
AXIN2	axis-inhibition protein 2
BLISS	Balanced Latent Integration with Stability Selection (this work)
BRICS	Breaking of Retrosynthetically Interesting Chemical Substructures
bRo5	beyond rule of five
CCND1	cyclin D1 gene
CDK4/6	cyclin-dependent kinase 4 and 6
CMS	consensus molecular subtypes (of colorectal cancer)
CNV	copy-number variation
CpG	cytosine–phosphate–guanine dinucleotide
CRBN	cereblon (E3 ligase substrate receptor)
CSV	comma-separated values (file format)
DC_5__0_	drug concentration causing 50% degradation
Dmax	maximum percent degradation
DCAF1	DDB1 and CUL4-associated factor 1
DNA	deoxyribonucleic acid
donorHB	QikProp predicted hydrogen-bond donor count
ETKDGv3	Experimental-Torsion Knowledge Distance Geometry, version 3
FDR	false-discovery rate
GDP	guanosine-5′-diphosphate
GELU	Gaussian Error Linear Unit
Glide SP	Glide Standard Precision (Schrödinger docking mode)
GPU	graphics processing unit
hERG	human Ether-à-go-go-Related Gene (potassium channel)
HOA	human oral absorption (qualitative class)
%HOA	QikProp predicted percent human oral absorption
IC_5__0_	concentration causing 50% inhibition
IP(eV)	QikProp predicted ionisation potential (electronvolts)
KL	Kullback–Leibler (divergence)
KLHDC2	kelch domain-containing protein 2
LEF1	lymphoid-enhancer-binding factor 1
LigPrep	Schrödinger ligand preparation utility
MAPK	mitogen-activated protein kinase
MDCK	Madin–Darby canine kidney (cell line)
#metab	QikProp predicted number of likely metabolic reactions
miRNA	microRNA
MM-GBSA	molecular mechanics combined with the generalised-Born/surface-area implicit-solvent model
MMFF94s	Merck Molecular Force Field 94, static variant
MOFA	multi-omics factor analysis
mRNA	messenger ribonucleic acid
MSI(-H)	microsatellite instability (high)
MSigDB	Molecular Signatures Database
MSS	microsatellite stable
mTOR	mechanistic (mammalian) target of rapamycin
MW	molecular weight
MYC	MYC proto-oncogene
#NandO	QikProp count of nitrogen + oxygen atoms
OPLS_2005/OPLS4	Optimised Potentials for Liquid Simulations force fields
PDB	Protein Data Bank
PEG	polyethylene glycol
PI3K	phosphoinositide 3-kinase
pKa	negative base-10 logarithm of the acid dissociation constant
PROPKA	empirical pKa prediction program
PROTAC	proteolysis-targeting chimera
PROTAC-DB	PROTAC database (Liu et al. [55], NAR 2025)
PSA	polar surface area (Å^2^)
QPlogBB	QikProp predicted log(brain/blood partition coefficient)
QPlogHERG	QikProp predicted log(IC_5__0_) for hERG K^+^ channel blockade
QPlogKhsa	QikProp predicted log(human serum albumin binding constant)
QPlogKp	QikProp predicted skin permeability (log cm h^−1^)
QPlogPC16	QikProp predicted log(hexadecane/gas partition coefficient)
QPlogPo/w	QikProp predicted log(octanol/water partition coefficient)
QPlogPoct	QikProp predicted log(octanol/gas partition coefficient)
QPlogPw	QikProp predicted log(water/gas partition coefficient)
QPlogS	QikProp predicted log(aqueous solubility, mol L^−1^)
QPPCaco	QikProp predicted Caco-2 cell permeability (nm s^−1^)
QPPMDCK	QikProp predicted MDCK cell permeability (nm s^−1^)
QPpolrz	QikProp predicted molecular polarisability (Å^3^)
RDKit	open-source cheminformatics toolkit (rdkit.org)
#ringatoms	QikProp count of atoms in rings
RMSD	root-mean-square deviation
ROC	receiver-operating-characteristic
#rotor	QikProp count of rotatable bonds
RPPA	reverse-phase protein array
SASA	solvent-accessible surface area (Å^2^)
SDF	structure-data file (format)
SMARTS	SMILES Arbitrary Target Specification (substructure language)
SMILES	Simplified Molecular-Input Line-Entry System
SNF	similarity network fusion
#stars	QikProp structural alerts (descriptors outside the 95% range)
Super.FELT	supervised feature-extraction learning using triplet loss [26]
TCGA	The Cancer Genome Atlas
TROP2	trophoblast cell-surface antigen 2
USR	Ultrafast Shape Recognition
USRCAT	USR with Chemical Features (atom-type-augmented shape descriptor)
VCB	VHL / Elongin-C / Elongin-B (E3-ligase complex)
VHL	von Hippel–Lindau (E3 ligase substrate receptor)
VSGB	Variable-dielectric Surface Generalised-Born (Schrödinger implicit-solvent model)
XGBoost	extreme gradient boosting
ΔG_bind	Gibbs free energy of binding (kcal mol^−1^)
