# Supplementary material for: AI-Driven Multi-Omics Integration of Synthetic Colon Adenocarcinoma for Cluster-Guided PROTAC Candidate Design Targeting KRASG12D
Source: Int J Mol Sci. 2026 Aug 22;27(17):7511. doi: 10.3390/ijms27177511 (PMC13565430; doi:10.3390/ijms27177511)
Supplement: Supplementary file 1 [file ijms-27-07511-s001.zip › ijms-4453767-Figures S1-S5.pdf]

Supplementary Materials

AI-Driven Multi-Omics Integration of Synthetic Colon Adenocarcinoma for Cluster-Guided PROTAC Candidate Design Targeting KRASG12D

Khaled M. Elamin [1], Sara Mustafa Idris Elbashir [1] and Ishag Adam [2,*]

[1] Graduate School of Pharmaceutical Sciences, Kumamoto University, Chuo-ku, Kumamoto 862-0973, Japan; khaled@kumamoto-u.ac.jp (K.M.E.); sarmah22807@kumamoto-u.ac.jp (S.M.I.E.)
[2] Department of Obstetrics and Gynecology, College of Medicine, Qassim University, Buraydah 51452, Saudi Arabia
* Correspondence: ia.ahmed@qu.edu.sa

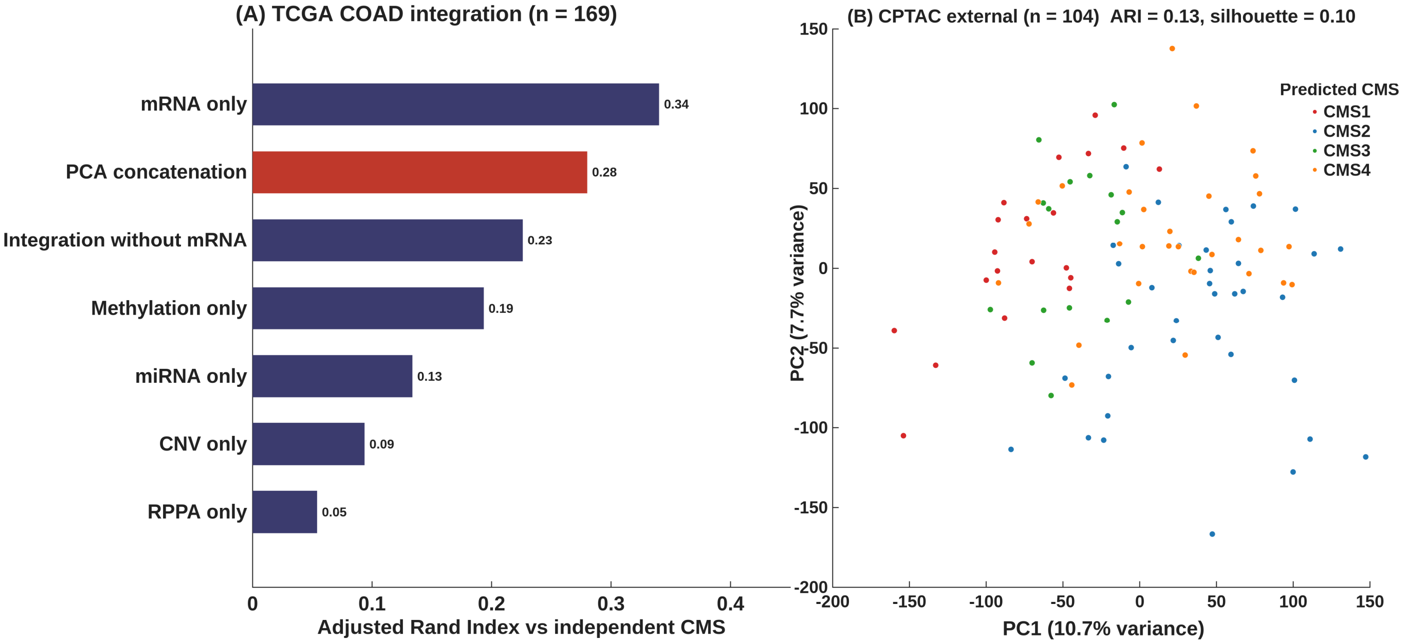

Figure S1. Multi-omics integration: TCGA discovery and CPTAC external validation. (**A**) Adjusted Rand Index between unsupervised clusters and independent CMScaller labels (n = 169 classified tumors) for each single layer and for five-layer integration; expression alone gives the highest agreement and concatenation does not exceed it. (**B**) Independent CPTAC external cohort (n = 104), integration embedding colored by predicted Consensus Molecular Subtype; external agreement Adjusted Rand Index 0.13, cluster silhouette 0.10, consistent with the modest TCGA result.

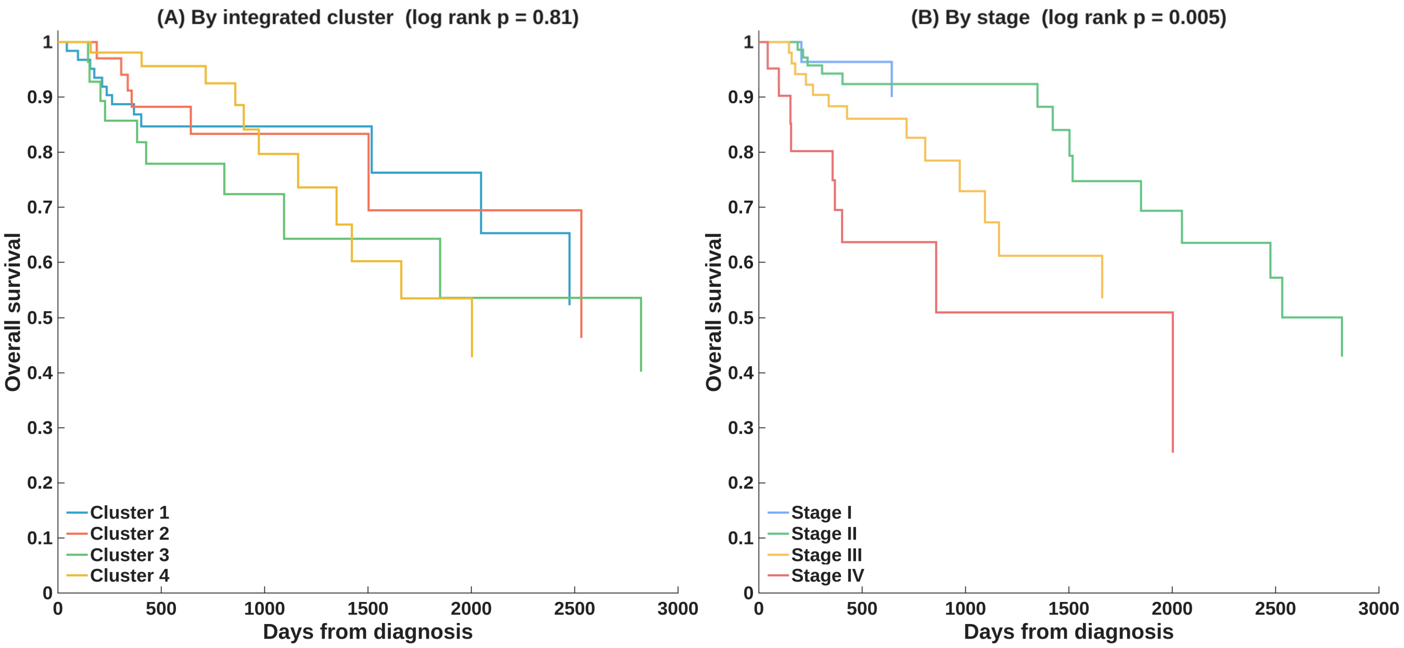

Figure S2. Real survival analysis. Kaplan Meier overall survival by (**A**) integrated molecular cluster (log-rank $p = 0.81$, not significant) and by (**B**) stage (log-rank $p = 0.005$; stage IV worst) in the real TCGA cohort.

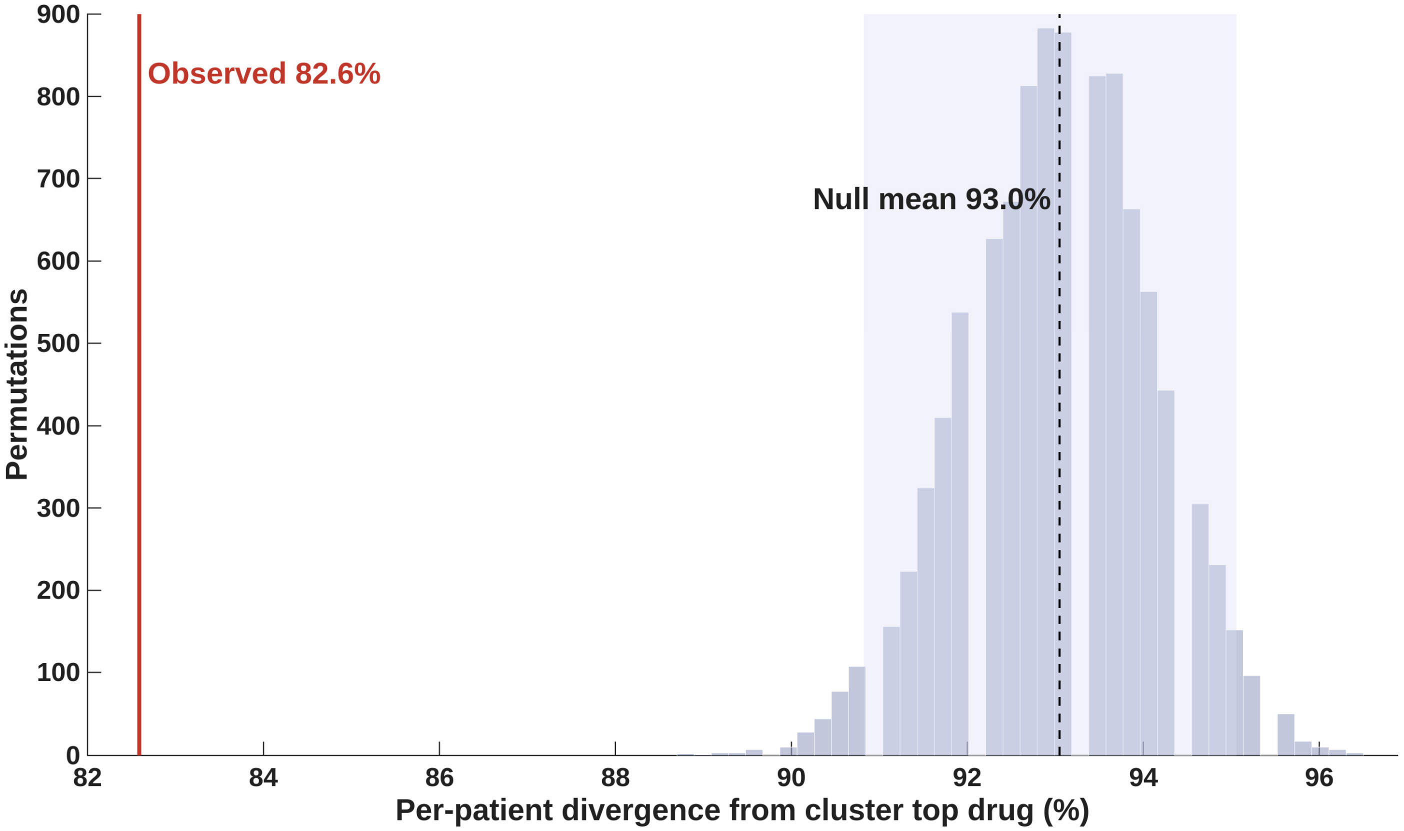

Figure S3. Label-permutation null model for patient-to-cluster drug-ranking concordance. Distribution of divergence under 10,000 label permutations. The observed value (82.6%) lies far below the null mean (93.0%), $p < 0.0001$.

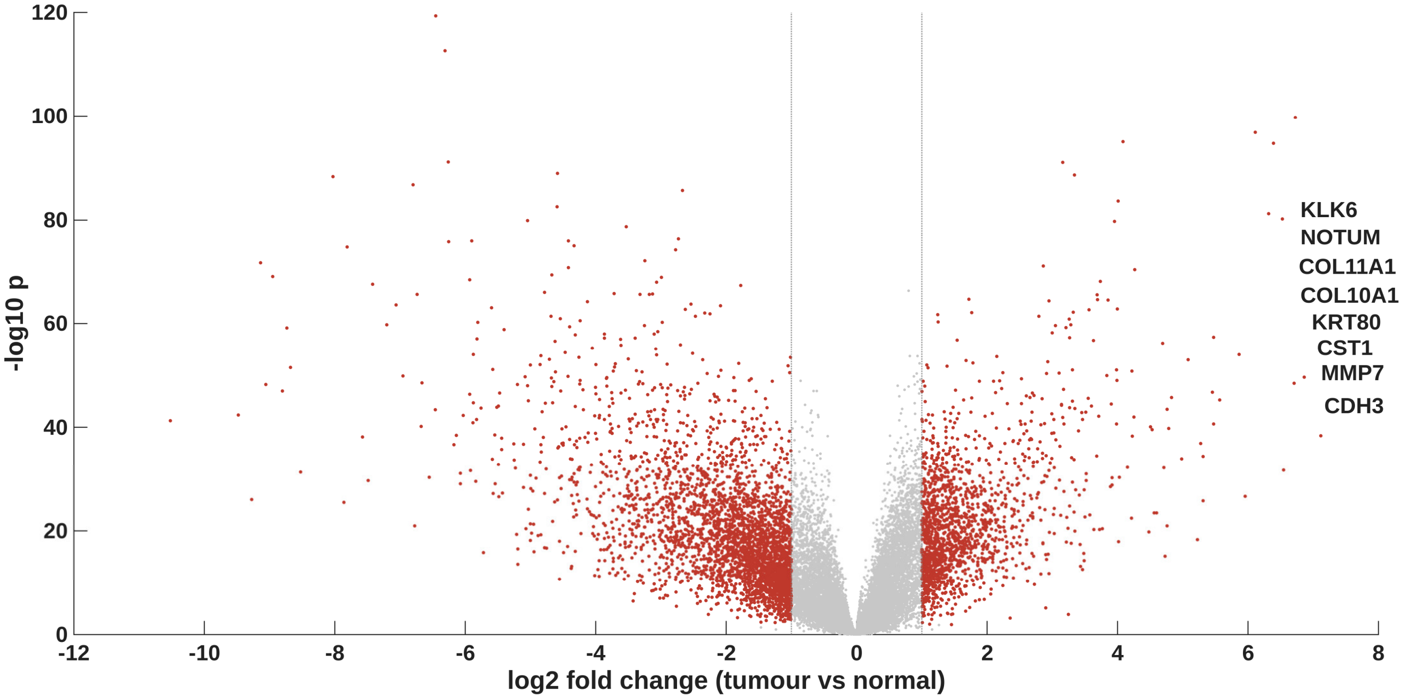

Figure S4. Real differential expression. Volcano plot of tumor versus normal expression in the real cohort; 4809 genes are significant at false discovery rate < 0.05 and absolute log2 fold change > 1.

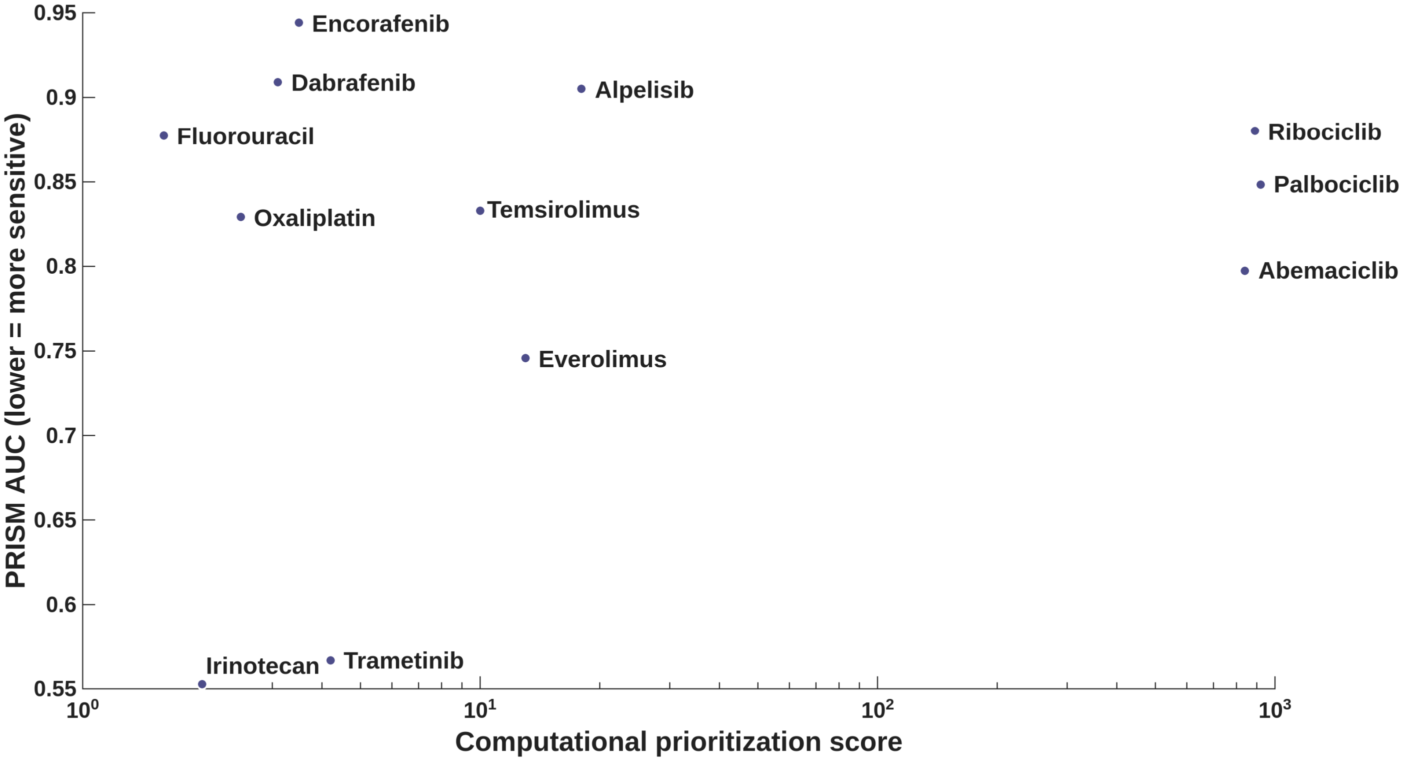

Figure S5. Pharmacogenomic validation against PRISM. Computational prioritization score versus real PRISM sensitivity (area under the curve; lower is more sensitive) in 38 colorectal cell lines for the 12 overlapping small molecules; no significant correlation (Spearman rho = -0.09, p = 0.78).
