# Supplementary material for: AI-Driven Multi-Omics Integration of Synthetic Colon Adenocarcinoma for Cluster-Guided PROTAC Candidate Design Targeting KRASG12D
Source: Int J Mol Sci. 2026 Aug 22;27(17):7511. doi: 10.3390/ijms27177511 (PMC13565430; doi:10.3390/ijms27177511)
Supplement: Supplementary file 1 [file ijms-27-07511-s001.zip › ijms-4453767-Tables S1 and S2.pdf]

Supplementary Table S1. Glide standard precision docking scores, Prime MM GBSA binding free energies, 3D shape similarity to the ASP 3082 bound conformation, and physicochemical descriptors of the top three novel KRAS G12D PROTAC chimeras (ranked by MM GBSA ΔG_bind) and the co crystallised clinical reference. All energies in kcal per mole; Phase Shape Similarity (r_phase_Shape_Sim) is dimensionless.

Compound	Docking Score (kcal/mol)	MM GBSA ΔG_bind (kcal/mol)	Shape Sim	MW (Da)	Heavy Atoms	Rot Bonds
**ASP 3082 (reference)**	−17.50	−165.65	1.000	1117.3	81	21
**NOVEL 00489 (lead)**	−16.54	−171.55	0.299	1117.4	80	21
**NOVEL 00894**	−14.01	−161.66	0.307	1190.3	87	23
**NOVEL 00977**	−14.26	−138.13	0.303	1231.3	89	23

Supplementary Table S2. Comprehensive ADMET descriptors computed by QikProp for the ASP 3082 clinical reference and the top three novel KRAS G12D PROTAC chimeras. Column 1: descriptor abbreviation; column 2: full name; column 3: recommended QikProp acceptable range; columns 4–7: descriptor values.

Abbrev.	Full Name	Acceptable Range	ASP 3082	N 00489	N 00894	N 00977
**mol MW**	Molecular weight (Da)	130–725 (Lipinski ≤ 500)	1117.3	1117.4	1190.3	1231.3
**#stars**	QikProp structural alerts	0–5 (lower = more drug like)	15	14	15	16
**dipole**	Dipole moment (Debye)	1.0–12.5	2.31	8.17	8.56	12.69
**SASA**	Solvent accessible surface area (Å^2)	300–1000	1616.0	1618.3	1673.5	1667.2
**volume**	Molecular volume (Å^3)	500–2000	3207.2	3234.7	3328.0	3337.9
**donorHB**	Hydrogen bond donor count	0.0–6.0	5.00	3.00	4.00	4.00
**accptHB**	Hydrogen bond acceptor count	2.0–20.0	20.35	23.10	18.65	17.65
**QPpolrz**	Predicted polarisability (Å^3)	13.0–70.0	116.4	115.4	120.1	120.2
**QPlogPo/w**	Predicted log(octanol/water)	−2.0 to 6.5	7.01	6.61	10.05	11.63
**QPlogS**	Predicted aqueous solubility (log mol/L)	−6.5 to 0.5	−12.71	−10.21	−15.18	−15.95
**QPlogHERG**	Predicted log(IC50) for hERG blockade	> −5 (low risk)	−7.07	−8.40	−10.03	−9.80
**QPPCaco**	Predicted Caco 2 permeability (nm/s)	< 25 poor; > 500 great	2.28	5.61	5.09	45.56
**QPlogBB**	Predicted log(brain/blood)	−3.0 to 1.2	−4.39	−2.64	−4.40	−2.48
**QPPMDCK**	Predicted MDCK permeability (nm/s)	< 25 poor; > 500 great	3.77	3.63	5.42	235.10
**QPlogKp**	Predicted skin permeability (log cm/h)	−8.0 to −1.0	−6.18	−6.82	−5.69	−3.96
**#metab**	Predicted metabolic reactions	1–8	12	10	11	13
**QPlogKhsa**	Predicted log(HSA binding)	−1.5 to 1.5	1.50	1.35	2.80	3.04
**HOA**	Oral absorption qualitative class	≥ 2 acceptable	1	1	1	1
**%HOA**	Predicted percent human oral absorption	> 80 high; 25–80 medium; < 25 low	35.52	40.19	59.56	85.86
**PSA**	Polar surface area (Å^2)	7–200 (oral); ≤ 140 ideal	226.69	205.21	241.08	210.90
**#NandO**	Number of N + O atoms	≤ 10 (Lipinski)	19	19	19	19
**#Lipinski**	Lipinski violations	0–1	3	3	3	3
**#Jorgensen**	Jorgensen violations	0–1	3	3	3	2
**#rotor**	Number of rotatable bonds	0–15	15	16	17	17
**#ringatoms**	Number of ring atoms	—	62	59	65	65
**IP(eV)**	Predicted ionisation potential (eV)	7.9–10.5	8.72	8.52	8.62	8.60
